# Endothelial loss of Fzd5 stimulates PKC/Ets1-mediated transcription of Angpt2 and Flt1

**DOI:** 10.1007/s10456-018-9625-6

**Published:** 2018-05-29

**Authors:** Maarten M. Brandt, Christian G. M. van Dijk, Ihsan Chrifi, Heleen M. Kool, Petra E. Bürgisser, Laura Louzao-Martinez, Jiayi Pei, Robbert J. Rottier, Marianne C. Verhaar, Dirk J. Duncker, Caroline Cheng

**Affiliations:** 1 Experimental Cardiology, Department of Cardiology, Thoraxcenter, Erasmus University Medical Center, Rotterdam, The Netherlands; 2 Department of Nephrology and Hypertension, Division of Internal Medicine and Dermatology, University Medical Center Utrecht, Utrecht, The Netherlands; 3 Department of Pediatric Surgery of the Erasmus Medical Center, Sophia Children’s Hospital, Rotterdam, The Netherlands

**Keywords:** Endothelial cells, Angiogenesis, Fzd5, Wnt signaling

## Abstract

**Aims:**

Formation of a functional vascular system is essential and its formation is a highly regulated process initiated during embryogenesis, which continues to play important roles throughout life in both health and disease. In previous studies, Fzd5 was shown to be critically involved in this process and here we investigated the molecular mechanism by which endothelial loss of this receptor attenuates angiogenesis.

**Methods and results:**

Using short interference RNA-mediated loss-of-function assays, the function and mechanism of signaling via Fzd5 was studied in human endothelial cells (ECs). Our findings indicate that Fzd5 signaling promotes neovessel formation in vitro in a collagen matrix-based 3D co-culture of primary vascular cells. Silencing of Fzd5 reduced EC proliferation, as a result of G_0_/G_1_ cell cycle arrest, and decreased cell migration. Furthermore, Fzd5 knockdown resulted in enhanced expression of the factors Angpt2 and Flt1, which are mainly known for their destabilizing effects on the vasculature. In Fzd5-silenced ECs, Angpt2 and Flt1 upregulation was induced by enhanced PKC signaling, without the involvement of canonical Wnt signaling, non-canonical Wnt/Ca^2+^-mediated activation of NFAT, and non-canonical Wnt/PCP-mediated activation of JNK. We demonstrated that PKC-induced transcription of Angpt2 and Flt1 involved the transcription factor Ets1.

**Conclusions:**

The current study demonstrates a pro-angiogenic role of Fzd5, which was shown to be involved in endothelial tubule formation, cell cycle progression and migration, and partly does so by repression of PKC/Ets1-mediated transcription of Flt1 and Angpt2.

**Electronic supplementary material:**

The online version of this article (10.1007/s10456-018-9625-6) contains supplementary material, which is available to authorized users.

## Introduction

New formation of blood vessels from pre-existing vessels, a process called angiogenesis, is a critical step in embryogenesis and continues to play important roles throughout life in both health and disease [1]. It is a dynamic process that is tightly regulated by a diverse range of signal transduction cascades, and imbalances in these pathways can be a causative or a progressive factor in many diseases [2].

Multiple studies suggest an important role for endothelial signal transduction via Frizzled (Fzd) receptors in angiogenesis [3–5]. The Fzd receptors belong to a family of 10 transmembrane receptors (Fzd1–10), which can initiate Fzd/Wnt canonical and non-canonical signaling upon binding with one of the 19 soluble Wnt ligands. Canonical Wnt signaling depends on Fzd receptor and LRP 5/6 co-activation, initiating Disheveled (Dvl) to stabilize β-catenin, followed by β-catenin-mediated transcriptional regulation [6–8]. In contrast, non-canonical Wnt signaling also involves Dvl, but proceeds via Wnt/Ca^2+^-mediated activation of nuclear factor of activated T-cells (NFAT) or Wnt/planar cell polarity (PCP)-mediated activation of c-JUN *N*-terminal Kinase (JNK) [6]. A potential link between Fzd5 and angiogenesis was previously demonstrated in Fzd5 full knockout mice [5]. Fzd5 silencing induced in utero death at approximately E10.5, which was associated with vascular defects in the placenta and yolk sac. Furthermore, isolated ECs from Fzd5-deficient mice showed a reduction in cell proliferation, which is crucial for neovessel formation. These findings suggest that Fzd5 can be an important regulator of angiogenesis. However, the exact type of endothelial Fzd5/Wnt signaling and the downstream molecular mechanism causal to the poor vascular phenotype in the absence of this receptor requires further in-depth evaluation.

Here, we studied the angiogenic potential of Fzd5 and investigated the signaling pathways that are mediated by Fzd5/Wnt signaling in human ECs. Our findings indicate that Wnt5a, which is endogenously expressed in ECs, binds and signals via Fzd5, but in the absence of this receptor triggers a poor angiogenic phenotype via an alternative signaling route. We demonstrated that Fzd5 is essential for neovessel formation in vitro in a collagen matrix-based 3D co-culture of primary human vascular cells. Silencing of Fzd5 reduced EC proliferation as a result of G_0_/G_1_ cell cycle arrest and decreased cell migration capacity. Furthermore, Fzd5 knockdown resulted in enhanced expression of the factors Angiopoietin 2 (Angpt2) and Fms-Related Tyrosine Kinase 1 (Flt1), which are mainly known for their destabilizing effects on the vasculature [9–11]. In Fzd5-silenced ECs, Angpt2 and Flt1 upregulation was induced by enhanced Protein Kinase C (PKC) signaling, without the involvement of canonical Wnt signaling, non-canonical Wnt/Ca^2+^-mediated activation of NFAT, and non-canonical Wnt/PCP-mediated activation of JNK. Further downstream, PKC-induced transcription of Angpt2 and Flt1 involved the transcription factor Protein C-Ets-1 (Ets1), as knockdown of both Fzd5 and Ets1 resulted in a marked repression of Angpt2 and Flt1 expression levels. In addition, silencing of Ets1 partially restored the impaired endothelial tubule formation capacity of Fzd5-silenced ECs.

## Methods

### Cell culture

Human umbilical vein endothelial cells (HUVECs; Lonza) and human brain vascular pericytes (Sciencell) were cultured on gelatin-coated plates in EGM2 medium (EBM2 medium supplemented with EGM2 bullet kit; Lonza, and 100 U/ml penicillin/streptomycin; Lonza) and DMEM (supplemented with 100 U/ml penicillin/streptomycin; Lonza, and 10% FCS; Lonza), respectively, in 5% CO_2_ at 37 °C. The experiments were performed with cells at passage 3–5. Lentivirus green fluorescent protein (GFP)-transduced HUVECs and lentivirus discosoma sp. red fluorescent protein (dsRED)-transduced pericytes were used at passages 5–7. HUVECs and GFP-labeled HUVECs were used from six different batches derived from pooled donors. Pericytes and dsRED-labeled pericytes were used from eight different batches derived from single donors. Fzd5, Ets1, and Wnt5a knockdown in HUVECs was achieved by cell transfection of a pool containing four targeting short interference RNA (siRNA) sequences, whereas PKC isoforms were knocked down with individual siRNA strands (Dharmacon), all in a final concentration of 100 nM. Control cells were either untreated or transfected with a pool of four non-targeting siRNA sequences (Dharmacon) in a final concentration of 100 nM. Target sequences are listed in Table 1. Inhibition of GSK3β, NFAT, JNK, and PKC activation was achieved with 20 µM LiCl (Sigma), 1 µM Cyclosporine A (CsA; Sigma), 20 µM SP600125 (Sigma), and 5, 10, and 20 nM staurosporine (CST), respectively. Phosphatase activity was inhibited with 50 nM Calyculin A. Free Ca^2+^-induced activation of NFAT-mediated transcription was achieved with 10 µM A23187. In experiments involving a serum starvation step, the cells were cultured for 24 h in EBM2.

**Table 1:** siRNA sequences used in cell culture

Target gene	Target sequence
Non-targeting	UGGUUUACAUGUCGACUAA
	UGGUUUACAUGUUGUGUGA
	UGGUUUACAUGUUUUCUGA
	UGGUUUACAUGUUUUCCUA
Fzd5	GCAUUGUGGUGGCCUGCUA
	GCACAUGCCCAACCAGUUC
	AAAUCACGGUGCCCAUGUG
	GAUCCGCAUCGGCAUCUUC
Ets1	AUAGAGAGCUACGAUAGUU
	GAAAUGAUGUCUCAAGCAU
	GUGAAACCAUAUCAAGUUA
	CAGAAUGACUACUUUGCUA
Wnt5a	GCCAAGGGCUCCUACGAGA
	GUUCAGAUGUCAGAAGUAU
	CAUCAAAGAAUGCCAGUAU
	GAAACUGUGCCACUUGUAU
PKCα	UAAGGAACCACAAGCAGUA
PKCδ	CCAUGUAUCCUGAGUGGAA
PKCε	GUGGAGACCUCAUGUUUCA
PKCη	GCACCUGUGUCGUCCAUAA

### Quantitative PCR and Western blot analysis

Total RNA was isolated using RNA mini kit (Bioline) and reversed transcribed into cDNA using iScript cDNA synthesis kit (Bioline). Gene expression was assessed by qPCR using SensiFast SYBR & Fluorescein kit (Bioline) and primers as listed in Table 2. Expression levels are relative to the housekeeping gene β-actin. For assessment of protein levels, cells were lysed in cold NP-40 lysis buffer (150 mM NaCl, 1.0% NP-40, 50 mM Tris, pH 8.0) supplemented with 1 mM β-glycerophosphate, 1 mM PMSF, 10 mM NaF, 1 mM NaOV, and protease inhibitor cocktail (Roche). Total protein concentration was quantified by Pierce® BCA Protein Assay Kit (Thermo Scientific) as a loading control. Lysates were denaturated in Laemmli buffer (60 mM Tris pH 6.8, 2% SDS, 10% glycerol, 5% β-mercaptoethanol, 0.01% bromophenol blue) at 90 °C for 5 min followed by electrophoresis on a 10% SDS-page gel (Biorad). Subsequently, proteins were transferred to a nitrocellulose membrane (Pierce) and incubated for 1 h in PBS with 5% non-fat milk, followed by incubation with rabbit anti-Fzd5 (Milipore), goat anti-β-actin (Abcam), rabbit anti-β-catenin, anti-non-phospho β-catenin and phospho-β-catenin (CST, validated in Supplemental Fig. 3A), rabbit anti-Angpt2 (Abcam), rabbit anti-JNK and phospho-JNK (CST, validated in Supplemental Fig. 4C), rabbit anti-JUN and phospho-JUN (CST, validated in Supplemental Fig. 4C), rabbit anti-Wnt5a (CST) rabbit anti-Dvl2 (CST) according to the manufacturer’s description. Protein bands were visualized with the Li-Cor detection system (Westburg). Levels of secreted Flt1 in cultured medium were assessed 72 h post-transfection using a Flt1 ELISA kit (R&D systems).

**Table 2:** Primer sequences used for (q)PCR

Gene	Sense primer sequence	Antisense primer sequence
Fzd1	GCCCTCCTACCTCAACTACCA	ACTGACCAAATGCCAATCCA
Fzd2	GCTTCCACCTTCTTCACTGTC	GCAGCCCTCCTTCTTGGT
Fzd3	CTTCCCTGTCGTAGGCTGTGT	GGGCTCCTTCAGTTGGTTCT
Fzd4	ATGAACTGACTGGCTTGTGCT	TGTCTTTGTCCCATCCTTTTG
Fzd5	TACCCAGCCTGTCGCTAAAC	AAAACCGTCCAAAGATAAACTGC
Fzd6	GCGGAGTGAAGGAAGGATTAG	TGAACAAGCAGAGATGTGGAA
Fzd7	CGCCTCTGTTCGTCTACCTCT	CTTGGTGCCGTCGTGTTT
Fzd8	GCCTATGGTGAGCGTGTCC	CTGGCTGAAAAAGGGGTTGT
Fzd9	CTGGTGCTGGGCAGTAGTTT	GCCAGAAGTCCATGTTGAGG
Fzd10	CCTTCATCCTCTCGGGCTTC	AGGCGTTCGTAAAAGTAGCAG
Wnt1	CAACAGCAGTGGCCGATGGTGG	CGGCCTGCCTCGTTGTTGTGAAG
Wnt2	GTCATGAACCAGGATGGCACA	TGTGTGCACATCCAGAGCTTC
Wnt2b	AAGATGGTGCCAACTTCACCG	CTGCCTTCTTGGGGGCTTTGC
Wnt3	GAGAGCCTCCCCGTCCACAG	CTGCCAGGAGTGTATTCGCATC
Wnt3a	CAGGAACTACGTGGAGATCATG	CCATCCCACCAAACTCGATGTC
Wnt4	GCTCTGACAACATCGCCTAC	CTTCTCTCCCGCACATCC
Wnt5a	GACCTGGTCTACATCGACCCC	GCAGCACCAGTGGAACTTGCA
Wnt5b	TGAAGGAGAAGTACGACAGC	CTCTTGAACTGGTTGTAGCC
Wnt6	TTATGGACCCTACCAGCAT	ATGTCCTGTTGCAGGATG
Wnt7a	GCCGTTCACGTGGAGCCTGTGCGTGC	AGCATCCTGCCAGGGAGCCCGCAGCT
Wnt7b	GATTCGGCCGCTGGAACTGCTC	TGGCCCACCTCGCGGAACTTAG
Wnt8a	CTGGTCAGTGAACAATTTCC	GTAGCACTTCTCAGCCTGTT
Wnt8b	GTCTTTTCACCTGTGTCCTC	AGGCTGCAGTTTCTAGTCAG
Wnt10a	CTGTTCTTCCTACTGCTGCT	ACACACACCTCCATCTGC
Wnt10b	GCACCACAGCGCCATCCTCAAG	GGGGTCTCGCTCACAGAAGTCAGGA
Wnt11	CACTGAACCAGACGCAACAC	CCTCTCTCCAGGTCAAGCAAA
Wnt14	ACAAGTATGAGACGGCACTC	AGAAGCTAGGCGAGTCATC
Wnt15	TGAAACTGCGCTATGACTC	GTGAGTCCTCCATGTACACC
Wnt16	GAGAGATGGAACTGCATGAT	GATGGGGAAATCTAGGAACT
Axin2	TTGAATGAAGAAGAGGAGTGGA	TCGGGAAATGAGGTAGAGACA
Ccnd1	GTCCATGCGGAAGATCGTCG	TCTCCTTCATCTTAGAGGCCACG
C-Myc	CACAGCAAACCTCCTCACAG	CGCCTCTTGACATTCTCCTC
Angpt1	GCTGAACGGTCACACAGAGA	CTTTCCCCCTCAAAGAAAGC
Angpt2	TTATCACAGCACCAGCAAGC	TTCGCGAGAACAAATGTGAG
VEGFa	AAGGAGGAGGGCAGAATCAT	ATCTGCATGGTGATGTTGGA
VEGFr2	AGCGATGGCCTCTTCTGTAA	ACACGACTCCATGTTGGTCA
Flt1	TGTCAATGTGAAACCCCAGA	GTCACACCTTGCTCCGGAAT
DSCR1	GAGGACGCATTCCAAATCAT	AGTCCCAAATGTCCTTGTGC
TF	TACTTGGCACGGGTCTTCTC	TGTCCGAGGTTTGTCTCCA
Ets1	GGAGCAGCCAGTCATCTTTC	GGTCCCGCACATAGTCCTT
PKCα	CGACTGGGAAAAACTGGAGA	ACTGGGGGTTGACATACGAG
PKCδ	ATTGCCGACTTTGGGATGT	TGAAGAAGGGGTGGATTTTG
PKCε	AAGCCACCCTTCAAACCAC	GGCATCAGGTCTTCACCAAA
PKCη	TCCCACACAAGTTCAGCATC	CCCAATCCCATTTCCTTCTT
MMP1	GATTCGGGGAGAAGTGATGTT	CGGGTAGAAGGGATTTGTG
Β-actin	TCCCTGGAGAAGAGCTACGA	AGCACTGTGTTGGCGTACAG

### 3D analysis of endothelial tubule formation

Twenty-four hours post siRNA transfection, GFP-labeled HUVECs were harvested and suspended with non-transfected dsRED-labeled pericytes in collagen as previously described by Stratman [12]. In summary, HUVECs and pericytes were mixed in a 5:1 ratio in EBM2 supplemented with Ascorbic Acid, Fibroblast Growth Factor, and 2% FCS from the EGM2 bullet kit. Additionally, C-X-C motif chemokine 12, Interleukin 3, and Stem Cell Factor were added in a concentration of 800 ng/ml (R&D systems). The cell mixture was suspended in bovine collagen (Gibco) with a final concentration of 2 mg/ml and pipetted in a 96-well plate. One hour of incubation in 5% CO_2_ at 37 °C was followed by the addition of 100 µl of the adjusted EBM2 medium on the collagen gels. The addition of recombinant human Angpt2 and Flt1 (R&D systems) was done 24 h post seeding in the collagen matrix, both in a final concentration of 1000 ng/ml. Forty-eight hours and 120 h post seeding, these co-cultures were imaged by fluorescence microscopy, followed by analysis of the number of junctions, the number of tubules, and the tubule length using AngioSys. At least three technical replicates were averaged per condition per independent replicate.

### Migration assay

Twenty-four hours post siRNA transfection, HUVECs were plated at a density of 0.5 × 10^5^ cells/well in an Oris™ Universal Cell migration Assembly Kit (Platypus Technologies) derived 96-well plate with cell seeding stoppers. Twenty-four hours post sub-culturing, the cell stoppers were removed and cells were allowed to migrate into the cell free region for 16 h in 5% CO_2_ at 37 °C. Subsequently, the cells were washed in PBS and stained by Calcein-AM followed by visualization using fluorescence microscopy. Wells in which cell seeding stoppers were not removed were used as a negative control. Results were analyzed by Clemex. At least three technical replicates were averaged per condition per independent replicate.

### Intracellular immunofluorescent staining

Forty-eight hours post siRNA transfection, HUVECs were seeded on gelatin-coated glass coverslips in 12-well plates at a density of 0.5 × 10^5^ cells/well (sub-confluent) and 3.5 × 10^5^ cells/well (confluent). Subsequently, cells adhered for 24 h followed by fixation for 15 min in 4% paraformaldehyde and blocking for 60 min in PBS with 5% bovine serum albumin (Sigma) and 0.3% Triton X-100 (Sigma). After blocking, coverslips were placed on droplets PBS containing 1% BSA, 0.3% Triton X-100, and rabbit anti-β-catenin antibody (CST), followed by incubation for 16 h in a humidified environment at 4 °C. Thereafter, coverslips were incubated on PBS with 1% BSA and 0.3% Triton X-100 containing an Alexa Fluor 594-labeled secondary antibody (Invitrogen) and phalloidin-rhodamine (Invitrogen) for 1 h at room temperature, finally followed by mounting the stained coverslips on vectashield with DAPI (Brunschwig). Coverslips were imaged by confocal microscopy.

### Proliferation, cell cycle assay, and apoptosis

Twenty-four hours post siRNA transfection, HUVECs were seeded in six-well plates at a density of 0.5 × 10^5^ cells/well. To study the effect of Fzd5 knockdown on proliferation, HUVECs were harvested 24, 48, and 72 h post sub-culturing and counted by flow cytometry. For analysis of cell cycle progression, cells were harvested 48 h post sub-culturing and fixated in 70% ethanol for 60 min on ice. Subsequently, cells were stained with PI and treated with RNAse (Sigma) for 30 min at 37 °C and analyzed by flow cytometry. Apoptosis was studied 72 h after transfection using an in situ cell death detection kit (Roche) as described by the manufacturer on 4% PFA fixated cells.

### Wnt5a adenovirus preparation, transduction, and stimulation

Recombinant adenoviruses were produced using the Gateway pAd/CMV/V5DEST vector and ViraPowerTM Adenoviral Expression System (Invitrogen), according to the manufacturer’s instructions. Briefly, the Wnt5a expression cassette was cloned from the pENTR™ 221 Wnt5a entry vector (Invitrogen) into pAd/CMV/V5-DEST expression vector (Invitrogen) via the LR-reaction II (invitrogen). After verification by DNA sequencing, the pAd/CMV plasmids were linearized by Pac1 restriction and subsequently transfected with Lipofectamine 2000 (Invitrogen) in HEK293A cells. Infected cells were harvested by the time 80% of the cells detached from plates followed by isolation of viral particles from crude viral lysate. HeLa cells were used to produce Wnt5a (or dsRED, referred to as adSHAM) by transduction with a calculated 5 viral particles per cell. Forty-eight hours post-transduction, HeLa cells were cultured for 24 h on EBM2, which eventually was used to stimulate serum-starved endothelium for 3 h.

### Statistical analysis

For each experiment, *N* represents the number of independent replicates. Statistical analysis was performed by GraphPad Prism using one-way ANOVA followed by post hoc Tukey’s test, unless stated otherwise. Results are expressed as mean ± SEM. Significance was assigned when *P* < 0.05 (two-tailed).

## Results

### Fzd5 siRNA induces a specific knockdown of endothelial Fzd5

The function of Fzd5 was studied in vitro using siRNA-mediated silencing in HUVECs, which were shown to express all Fzd receptors other than Fzd10 (Supplemental Fig. 1A), and Wnt2b, 3, 4, 5a, and 11 (Supplemental Fig. 1B). Both qPCR and Western blot analysis confirmed a significant loss of Fzd5 expression in cells treated with an siRNA pool specific for Fzd5, compared to untreated control cells and cells treated with a pool of non-targeting siRNA, referred to as siSHAM (Supplemental Fig. 1C,D). Although Fzd receptors share highly similar domains, knockdown of Fzd5 was specific. None of the other Fzd receptors were differentially expressed after treatment with Fzd5 siRNA, other than Fzd5 (Supplemental Fig. 1C).

### Wnt5a signals via endothelial Fzd5

**Fig. 1:**
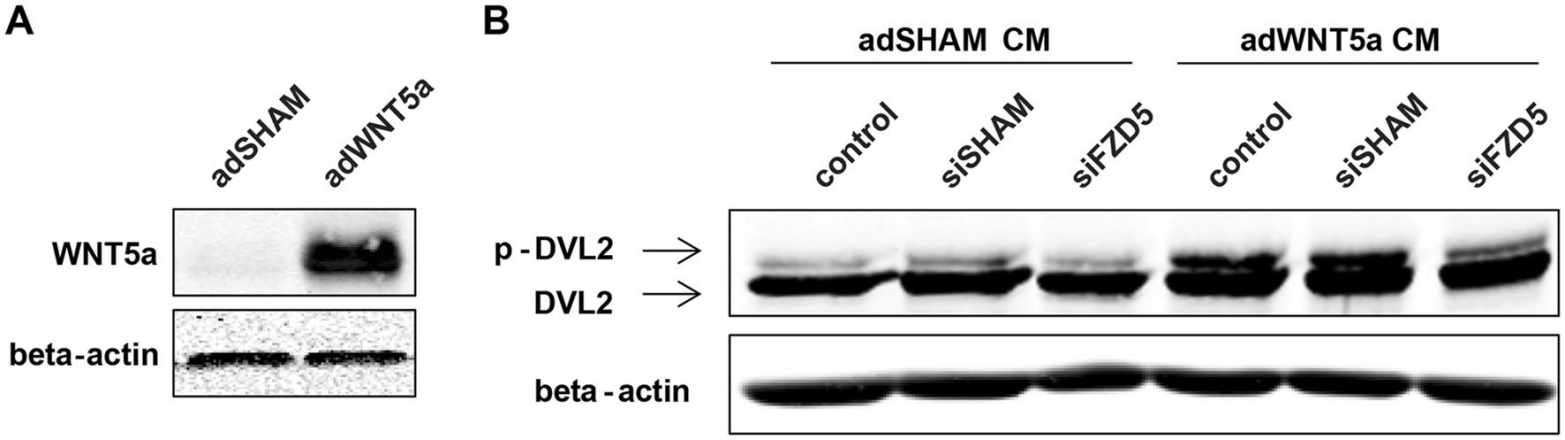
Wnt5a induced Fzd5-mediated Dvl activation in HUVECs. **a** Representative Western blot of adenoviral-based Wnt5a overexpression in HeLa cells, 72 h post-transduction. *N* = 4. **b** Representative Western blot of Dvl and phosphorylated Dvl in HUVECs after 3 h stimulation with cultured medium (CM) from HeLa cells overexpressing dsRED (adSHAM) or Wnt5a, 72 h post siRNA transfection in HUVECs. *N* = 6

Previous studies listed Wnt5a and Secreted Frizzled-Related Protein 2 (SFRP2) as most likely candidates to activate Fzd5-mediated signaling in ECs [13–15]. In contrast to SFRP2 [16], Wnt5a is endogenously expressed by HUVECs (Supplemental Fig. 1B). To address the potential signal capacities of this endogenously expressed Wnt5a as ligand for Fzd5, HeLa cells were transduced with an adenoviral overexpression plasmid for Wnt5a to produce cultured medium containing high levels of this Wnt ligand. HeLa cells were selected for this purpose over HUVECs as these cells were shown to have a more refined machinery to produce and secrete functional Wnt5a than HUVECs, as illustrated by enhanced mRNA expression of Wntless (WLS) and Porcupine (PORCN) (data not shown). Transduction with this overexpression vector (adWnt5a) led to a significant upregulation of Wnt5a compared to dsRED control transduced cells (adSHAM) (Fig. 1a). To assess whether Fzd5 was involved in transducing the signal of Wnt5a, cultured medium from transduced HeLa cells was applied to serum-starved HUVECs after which Dvl activation was monitored. Western blot analysis showed that Wnt5a strongly induced Dvl phosphorylation in untreated or non-targeting siRNA-treated HUVECs, however, this effect was blocked in the absence of Fzd5 (Fig. 1b), confirming the importance of endothelial Fzd5 in transducing Wnt5a signaling.

### Fzd5 expression is essential for endothelial proliferation, migration, and tubule formation

**Fig. 2:**
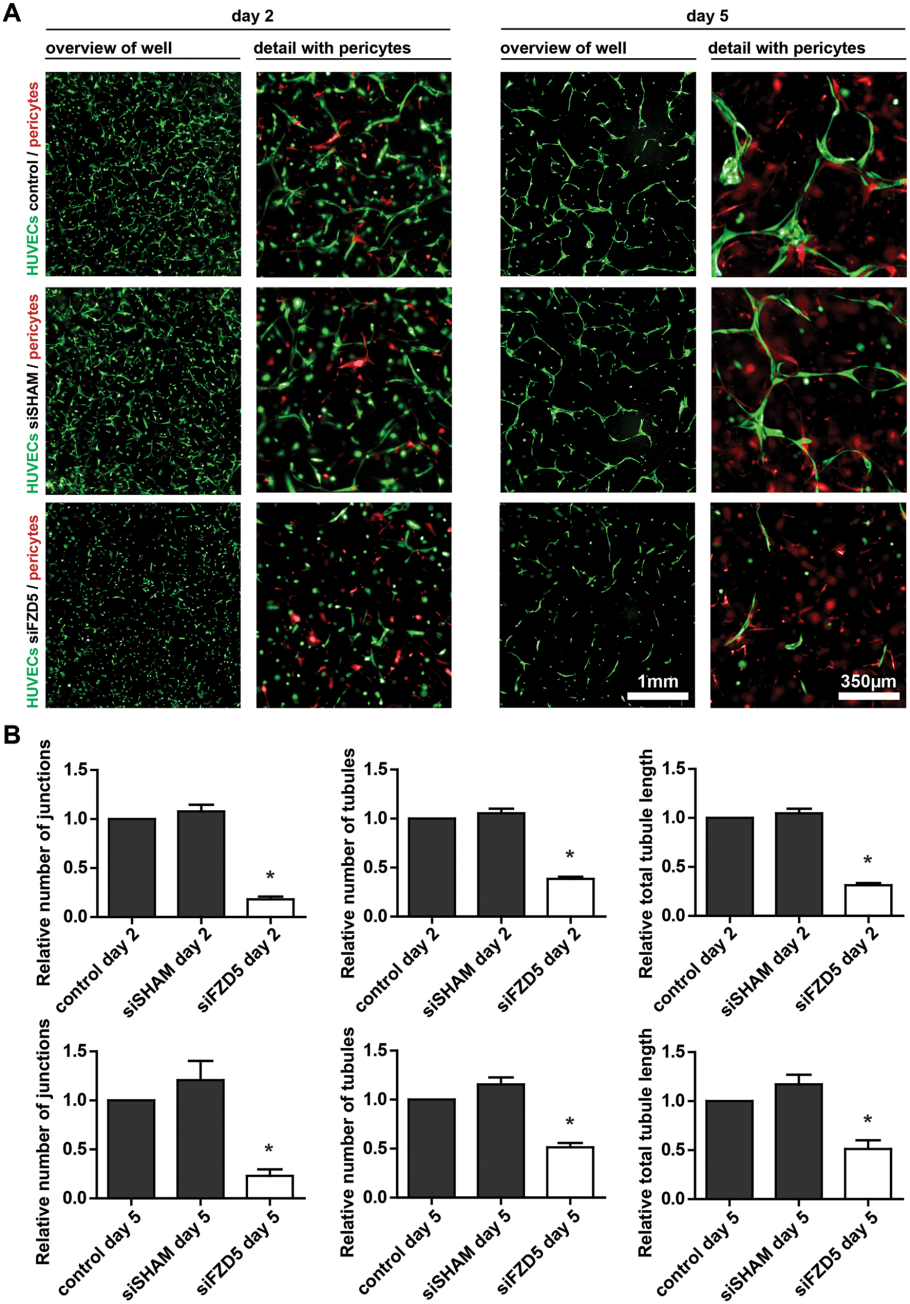
Fzd5 expression is crucial for vascular formation in vitro. **a** Representative fluorescent microscope images of GFP-labeled HUVECs (green) in co-culture with dsRED-labeled pericytes (red) in a 3D collagen matrix during vascular formation. Shown are the results at day 2 and 5 of non-transfected control, siSHAM, and siFzd5 conditions. Scale bar in the left columns represents 1 mm. Scale bar in the right columns represents 350 µm. **b** Bar graphs show the quantified results of the co-culture assay. Shown are the total tubule length, and the number of endothelial junctions and tubules relative to the control conditions, both after 2 and 5 days. *N* = 4, *P < 0.05 compared to control and siSHAM condition

The angiogenic capacities of these Fzd5-silenced HUVECs were evaluated in a well-validated in vitro 3D angiogenesis assay developed for studying formation of micro-capillary structures [12]. In this assay, HUVECs with GFP marker expression and dsRED-labeled pericytes directly interact in a collagen type I matrix environment, resulting in EC sprouting, tubule formation, and neovessel stabilization as a result of perivascular recruitment of pericytes. At day 5 post-seeding, well-defined, micro-capillaries with pericyte coverage can be observed. Imaging and quantification of the vascular structures were conducted at days 2 and 5. Endothelial knockdown of Fzd5 strongly impaired endothelial tubule formation (Fig. 2a). Quantification revealed a significant reduction in the total tubule length, the number of endothelial junctions, and the number of endothelial tubules, both after 2 and 5 days (Fig. 2b).

**Fig. 3:**
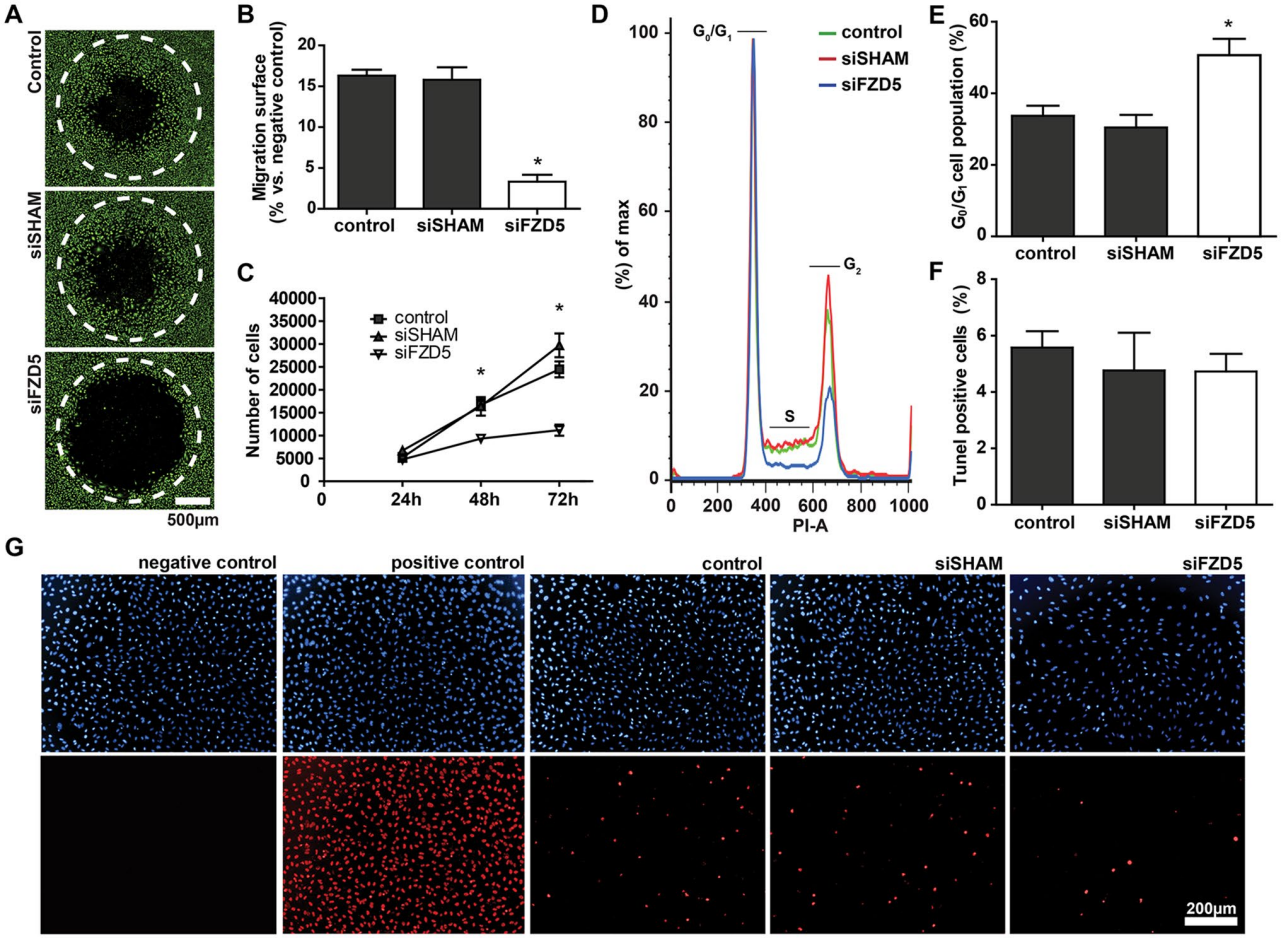
Endothelial knockdown of Fzd5 significantly inhibited EC migration and proliferation, but had no effect on apoptosis. **a** Representative fluorescent microscope images of Calcein-AM-labeled HUVECs (green) in a plug-stopper-based migration assay. Shown are the results of 16 h of migration of non-transfected control, siSHAM, and siFzd5 conditions. Scale bar represents 500 µm. Open migration areas produced by the plug-stopper before initiation of the assay are indicated by dotted lines. **b** Bar graph shows the quantified results of migration assay. Shown are the percentages of surface area within the dotted circle covered by HUVECs after 16 h of migration. *N* = 4, *P < 0.05 compared to control and siSHAM condition. **c** ECs expansion at 24, 48, and 72 h post seeding in similar densities, as quantified by flow cytometry. *N* = 3, *P < 0.05 compared to control and siSHAM condition (two-way ANOVA followed by Bonferroni post hoc test). **d** Representative histogram of flow cytometric analysis of PI-based DNA staining showing the distribution of cells over the cell cycle in the different groups at 48 h post-transfection. **e** Quantified results of cell cycle analysis. Percentage of cells in G_0_/G_1_ phase is shown. *N* = 3, *P < 0.05 compared to control and siSHAM condition. **f** Quantified results of TUNEL staining. Percentage TUNEL-positive cells of total number of cell is shown, 72 h post-transfection of control, siSHAM, and siFzd5 conditions. *N* = 3, no significance. **g** Representative fluorescent microscope images of DAPI-based nuclei staining in HUVECs (blue, upper row) and TUNEL staining of the same cells (red, lower row). Positive control was treated with DNAse solution. Scale bar represents 200 µm

To get a better insight in the causative factor for this poor vascular phenotype, the migration and proliferation capacities of Fzd5-silenced ECs were studied. A plug-stopper-based migration assay was performed to analyze the effects of Fzd5 knockdown on endothelial mobility. Knockdown of Fzd5 significantly inhibited the migration of ECs towards the open cell-devoid area compared to untreated and non-targeting siRNA-treated ECs (Fig. 3a, b). In addition, knockdown of Fzd5 significantly reduced cell numbers compared to control and siSHAM condition (Fig. 3c). To clarify whether this was a result of impaired cell proliferation or increased apoptosis, cell cycle progression was analyzed in a cell cycle assay in which total DNA was stained with PI, followed by flow cytometry. A strong increase of cells in the G_0_/G_1_ phase of the cell cycle was observed after knockdown of Fzd5, indicative of a cell cycle arrest (Fig. 3d, e). For apoptosis analysis, a terminal deoxynucleotidyl transferase dUTP nick end labeling (TUNEL)-based detection staining was used. Although seeded in similar densities, Fzd5 knockdown led to a significant reduction of nuclei per image field. However, the relative number of TUNEL positive nuclei in the Fzd5 knockdown condition was similar when compared to control and siSHAM condition, showing that the reduction of ECs in the Fzd5 knockdown condition is not related to increased apoptosis (Fig. 3f, g).

### Loss of Fzd5 does not interfere with endogenous canonical Wnt signaling

**Fig. 4:**
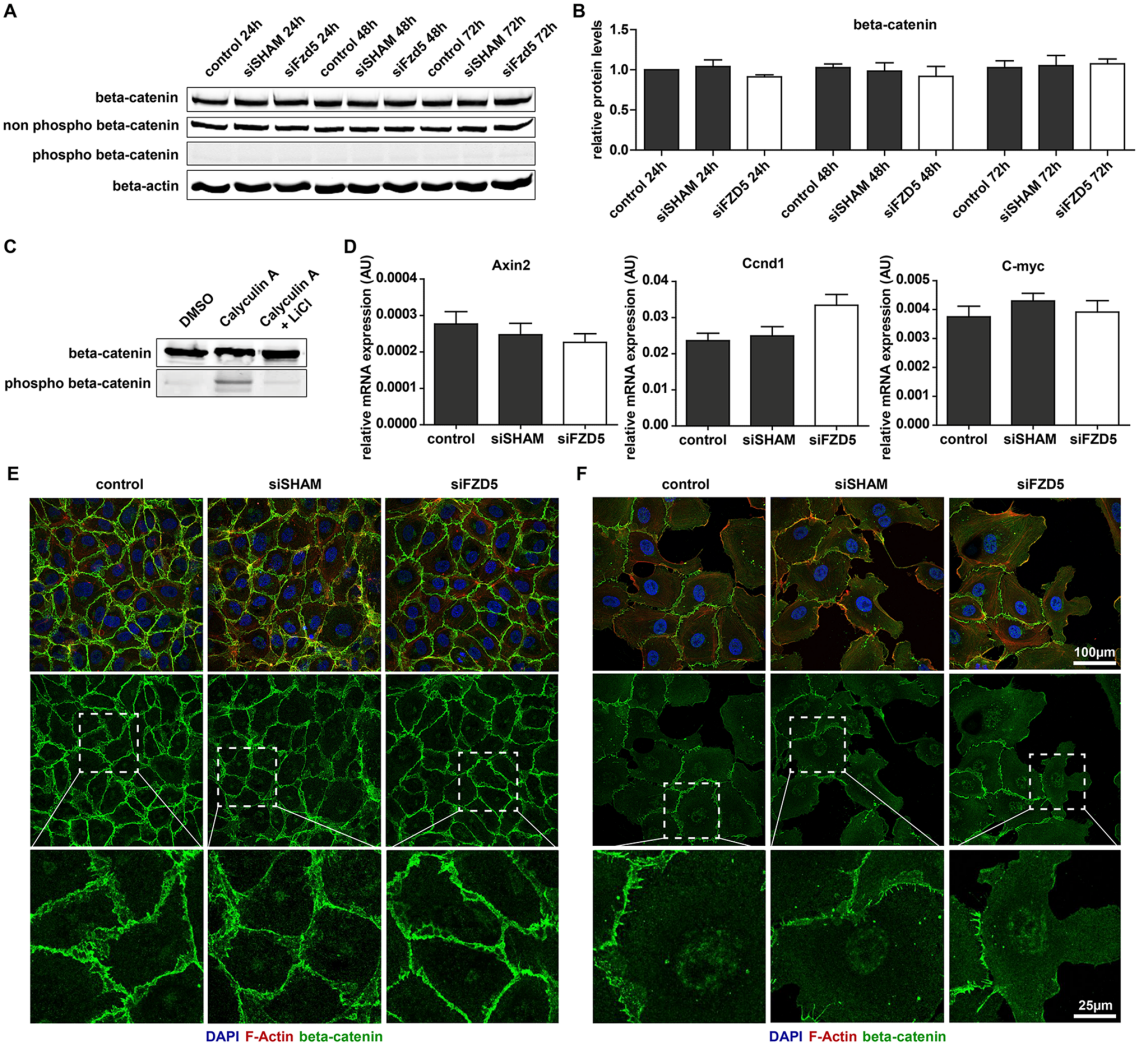
Fzd5 knockdown did not affect the canonical Wnt signaling pathway in ECs. **a** Representative Western blot result of total levels of β-catenin, non-phospho-β-catenin, phospho-β-catenin (ser33/37/thr41), and β-actin loading control, at different time points post-transfection. **b** Quantified results of β-catenin Western blot. Shown are β-catenin levels relative to β-actin loading control. *N* = 3, no significance. **c** Western blot result of total levels of β-catenin and phospho-β-catenin in response to treatment with the phosphatase inhibitor Calyculin A (50 nM) with and without a 30 min pretreatment of the GSK3β inhibitor LiCl (20 mM). **d** QPCR analysis of the mRNA expression levels of β-catenin target genes Axin2, Cyclin D1 (Ccnd1), and C-myc in different conditions 72 h post-transfection. *N* = 4, no significance. **e** Immunofluorescent staining β-catenin (green), F-actin (red), and DAPI (blue) in confluent and sub-confluent **f** HUVECs after knockdown of Fzd5. *N* = 3

To further dissect the molecular mechanism of endothelial Fzd5 signaling in angiogenesis, known Fzd/Wnt signaling pathways were studied. Downstream Fzd signaling occurs via the canonical Wnt signaling pathway, also known as the Wnt/β-catenin pathway, or by the less well described non-canonical Wnt signaling pathways. Activation of canonical Wnt signaling is characterized by an accumulation of cytoplasmic β-catenin, eventually resulting in nuclear translocation and subsequent expression of β-catenin-dependent target genes. To evaluate the effect of Fzd5 knockdown on the canonical Wnt signaling pathway, total levels of β-catenin, as well as phospho-β-catenin (ser33/37/thr41) and non-phospho-β-catenin (active) were examined 24, 48 and 72 h post-transfection by Western blot. Ser33/37/thr41 phosphorylation is induced by GSK3β and primes β-catenin for subsequent degradation, and could be indicative for a reduced activity of canonical Wnt signaling. Total β-catenin, as well as non-phospho-β-catenin (active) levels were unaffected by Fzd5 silencing, and non-phospho-β-catenin (ser33/37/thr41) was observed in all conditions (Fig. 4a, b), even though the antibody was capable of detecting GSK3β-induced β-catenin phosphorylation (Fig. 4c). Furthermore, expression levels of previously described endothelial target genes of β-catenin were studied using qPCR, but no differences were observed in the expression of Axin2, Ccnd1, and C-myc after knockdown of Fzd5 (Fig. 4d). An immunofluorescent staining, validated to detect cellular distribution of β-catenin (Supplemental Fig. 3B), was also performed on transfected ECs, as stable total levels of β-catenin found by Western blot did not deviate between cytoplasmic or nuclear localized β-catenin. In line with the other experiments focusing on β-catenin-mediated signaling, no differences in β-catenin localization were observed after knockdown of Fzd5, both in confluent and sub-confluent cells (Fig. 4e, f, respectively).

### Fzd5 knockdown induces the expression of several (anti-) angiogenic factors

**Fig. 5:**
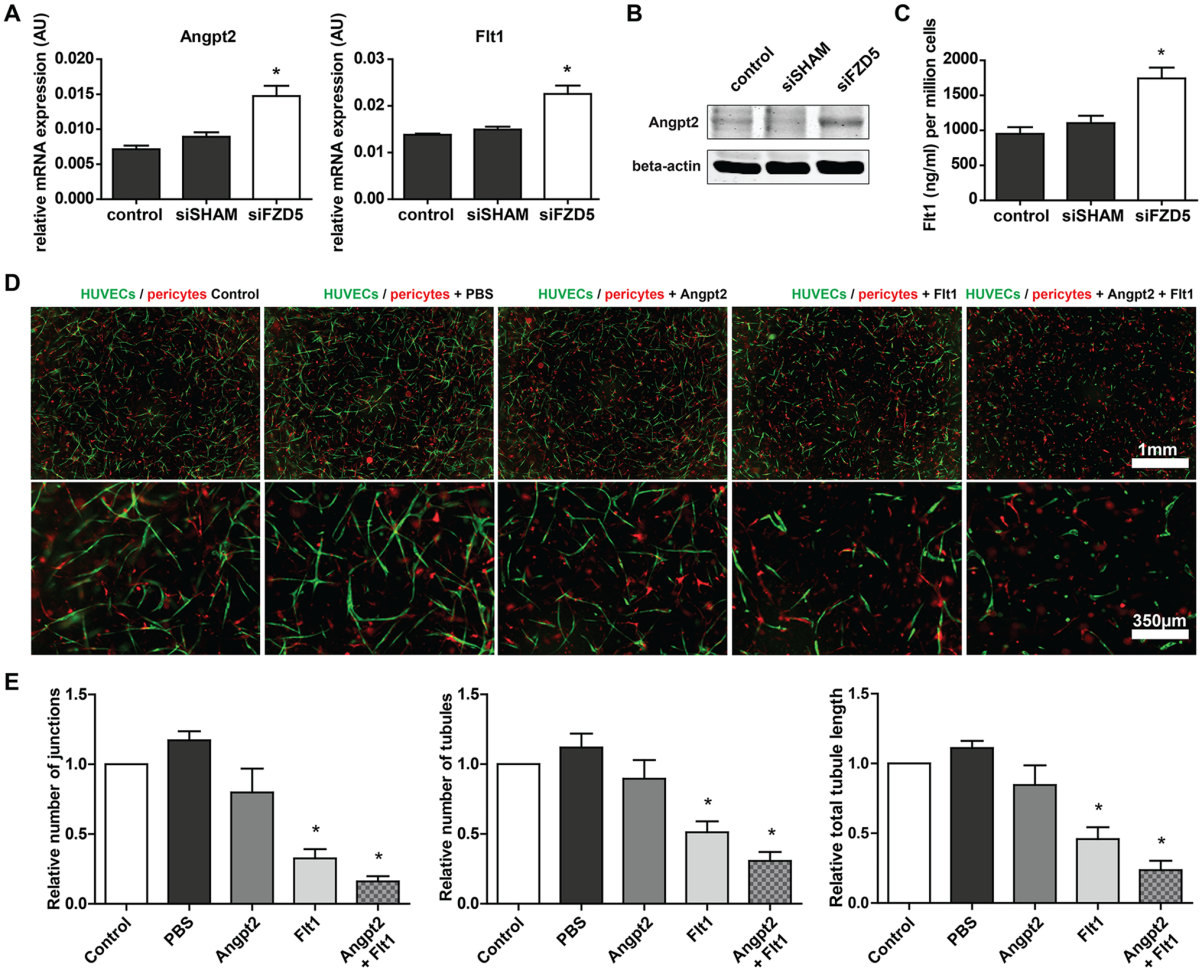
Fzd5 knockdown led to increased expression of vascular regression-associated genes Flt1 and Angpt2. **a** QPCR results of expression levels of Angpt2 and Flt1 in different conditions 72 h post-transfection. *N* = 11, *P < 0.05 compared to control and siSHAM condition. **b** Representative Western blot results of Angpt2 expression levels in the different conditions 72 h post-transfection. *N* = 3. **c** Enzyme-linked immunosorbent assay-based quantification of secreted Flt1 levels in cultured endothelial medium 72 h post-transfection. *N* = 8, *P < 0.05 compared to control and siSHAM condition. **d** Representative fluorescent microscope images of GFP-labeled HUVECs (green) in co-culture with dsRED-labeled pericytes (red) in a 3D collagen matrix during vascular formation. Shown are the results at day 5 of an untreated control, and after stimulation with PBS, Angpt2 (1000 ng/ml), Flt1 (1000 ng/ml), and Angpt2 + Flt1 (1000 ng/ml both). Scale bar in the upper row represent 1 mm, in the bottom row 350 µm. **e** Bar graphs show the quantified results of the co-culture assay. Shown are the total tubule length, and the number of endothelial junctions and tubules after 5 days. *N* = 4, *P < 0.05 compared to control and siSHAM condition

To further elucidate the anti-angiogenic phenotype observed after Fzd5 knockdown, expression levels of several important regulators of angiogenesis were analyzed. In contrast to what was previously reported [17], our findings in HUVECs indicate that expression of tissue factor (TF) is not positively regulated by Fzd5 signaling, as Fzd5 knockdown did not attenuate TF expression. In fact, TF was slightly upregulated in Fzd5-silenced HUVECs compared to untreated control cells, yet was statistically equal to non-targeting siRNA-treated HUVECs (Supplemental Fig. 2). Interestingly, vascular endothelial growth Factor A (VEGFa) decoy receptor Flt1, and the vascular destabilizing factor Angpt2 were significantly upregulated at both mRNA and protein level in HUVECs treated with Fzd5 siRNA when compared to untreated or non-targeting siRNA-treated HUVECs (Fig. 5a, c). Expression levels of VEGF receptor 2, VEGFa, as well as Angpt1 remained unaffected in the absence of Fzd5 (Supplemental Fig. 2). In line with previous findings of Lobov et al., combined addition of Flt1 and Angpt2 in the 3D co-culture system completely attenuated endothelial tubule formation (Fig. 5d, e) [9].

**Fig. 6:**
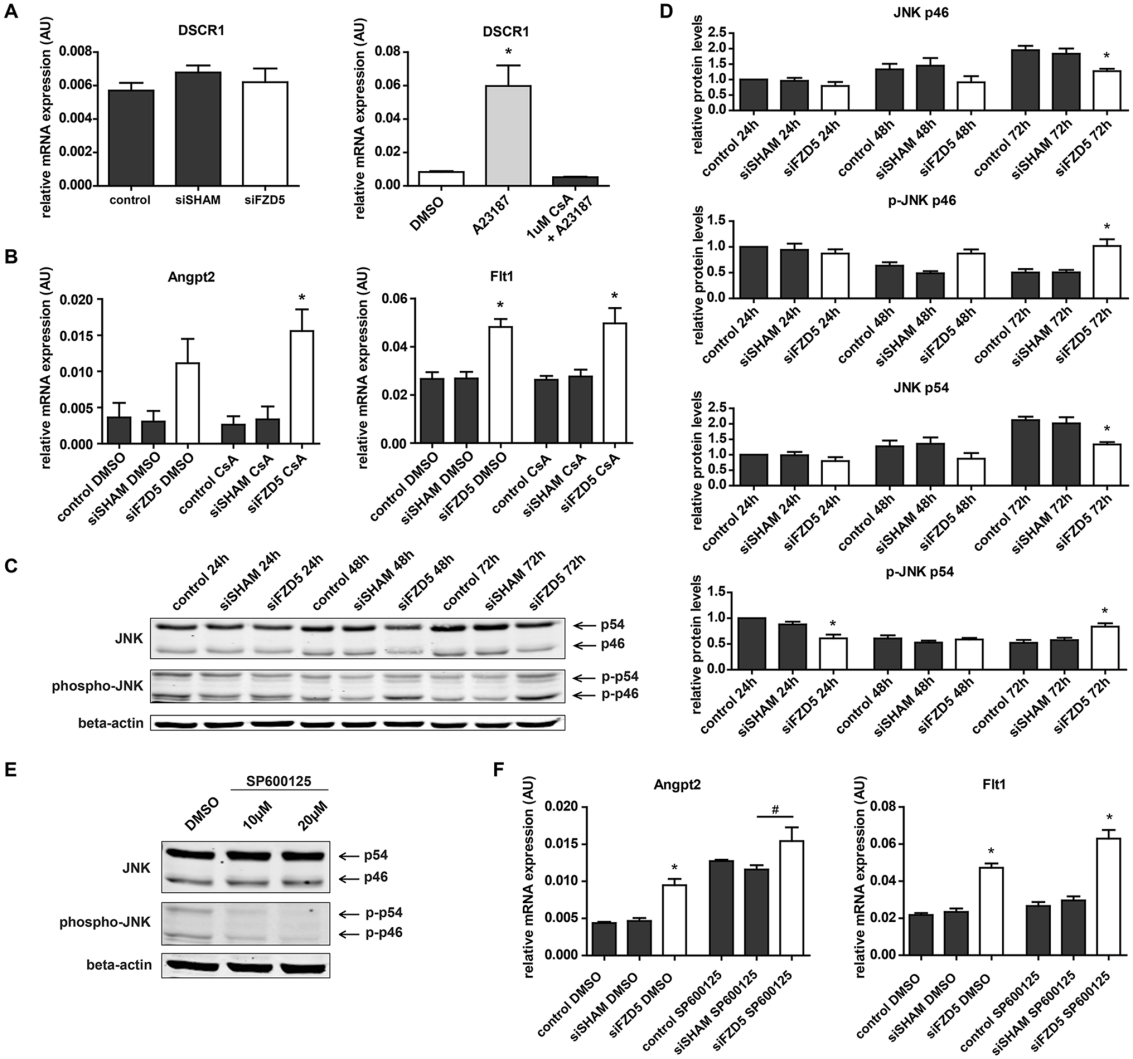
Fzd5 knockdown led to increased expression of vascular regression-associated genes Flt1 and Angpt2, independent of the non-canonical Wnt/Ca^2+^ and PCP pathways. **a** QPCR results of expression levels of NFAT target gene Dscr1 in the different conditions 72 h post-transfection and in response to ionophore A23187 (10 µM)-induced Ca^2+^ flux with and without NFAT inhibitor Cyclosporin A (CsA) (1 µM). *N* = 5, *P < 0.05 compared to control and siSHAM condition, and DMSO-treated and CsA + A23187-treated ECs, respectively. **b** Angpt2 and Flt1 mRNA expression levels in HUVECs in response to CsA, supplemented 48 h post-transfection. *N* = 4, *P < 0.05 compared to control and siSHAM condition (two-way ANOVA followed by Bonferroni post hoc test). **c** Representative Western blot of total JNK, phospho-JNK, and β-actin levels at different time points post-transfection. **d** Quantified results of JNK and phospho-JNK Western blot. Shown are individual (phospho) JNK isoform (p46 and p54) levels relative to β-actin loading control. *N* = 6, *P < 0.05 compared to control and siSHAM condition within one time comparison (24, 48 or 72 h). **e** Western blot of total JNK, phospho-JNK, and β-actin levels in response to different concentrations of JNK inhibitor SP600125 after 1 h. **f** QPCR analysis showing the effect of SP600125, supplemented 48 h post-transfection, on Flt1 and Angpt2 mRNA levels in the different conditions. *N* = 4, *P < 0.05 compared to control and siSHAM condition, ^#^*P* < 0.05 as indicated in graph (two-way ANOVA followed by Bonferroni post hoc test)

Knockdown of a Fzd receptor can not only attenuate signal transduction, but due to impaired inhibitory crosstalk between the individual pathways, or via alternative receptor binding by the Wnt ligand can also have a stimulatory effect [18, 19]. Since Fzd5 knockdown had no effect on the canonical Wnt signaling pathway, the described non-canonical Wnt/Ca^2+^ and PCP pathways were studied for their potential role in the upregulation of Angpt2 and Flt1. Activation of the Wnt/Ca^2+^ pathway could induce Flt1 and Angpt2 transcription, as stimulation of the Wnt/Ca^2+^ pathway leads to free Ca^2+^-induced activation of Calcineurin, which in turn could promote NFAT-mediated transcription by dephosphorylating NFAT [6]. The mRNA expression level of Down Syndrome Critical Region 1 (DSCR1) was evaluated to assess the potential link between Fzd5 knockdown and NFAT activation, as DSCR1 is a profound target gene of NFAT, involved in a feedback loop to fine-tune NFAT-mediated transcription [20, 21]. However, no correlation between endothelial knockdown of Fzd5 and DSCR1 upregulation was observed (Fig. 6a). The involvement of NFAT-mediated transcription was also evaluated by pharmacological inhibition of the Wnt/Ca^2+^ signaling cascade using the Calcineurin inhibitor Cyclosporine A (CsA). The effectiveness of CsA (1 µM) was confirmed by its ability to inhibit calcium ionophore (A23187)-induced transcription of DSCR1 as a result of free Ca^2+^-mediated NFAT activation in ECs (Fig. 6a). In line with the absence of DSCR1 upregulation in the Fzd5 knockdown condition, the upregulation of Flt1 and Angpt2 could not be linked to an increase of NFAT-mediated transcription in the Fzd5 knockdown condition, as CsA stimulation failed to reduce Angpt2 and Flt1 upregulation in Fzd5-silenced cells (Fig. 6b).

Besides activation of the Wnt/Ca^2+^ pathway, the PCP pathway could also stimulate the expression of Flt1 and Angpt2 via activation of the Wnt/PCP signaling cascade linked to downstream JNK-induced transcriptional activation of c-JUN [22, 23]. Activation of JNK/c-JUN-mediated transcription involves phosphorylation of JNK, which was slightly increased both 48 and 72 h post-transfection (Fig. 6c, d). JNK-mediated phosphorylation of c-JUN, however, was not observed (Supplemental Fig. 4A, B). Since JNK is a kinase with a broad spectrum of downstream substrates [24], the JNK inhibitor SP600125 was used to block activation of JNK to define whether the enhanced phosphorylation of JNK played a role in the upregulation of Flt1 and Angpt2. The effectiveness of SP600125 (20 µM) was confirmed by its ability to inhibit JNK phosphorylation in ECs (Fig. 6e). Treatment of HUVECs with SP600125 did not diminish Fzd5 silencing-induced upregulation of Flt1 and Angpt2 (Fig. 6f). In contrast, SP600125 treatment rather induced a general upregulation of Angpt2, indicating that activation of JNK was not causally related to the Fzd5 knockdown-mediated upregulation of both genes.

### Angpt2 and Flt1 upregulation is mediated via PKC and Ets1

**Fig. 7:**
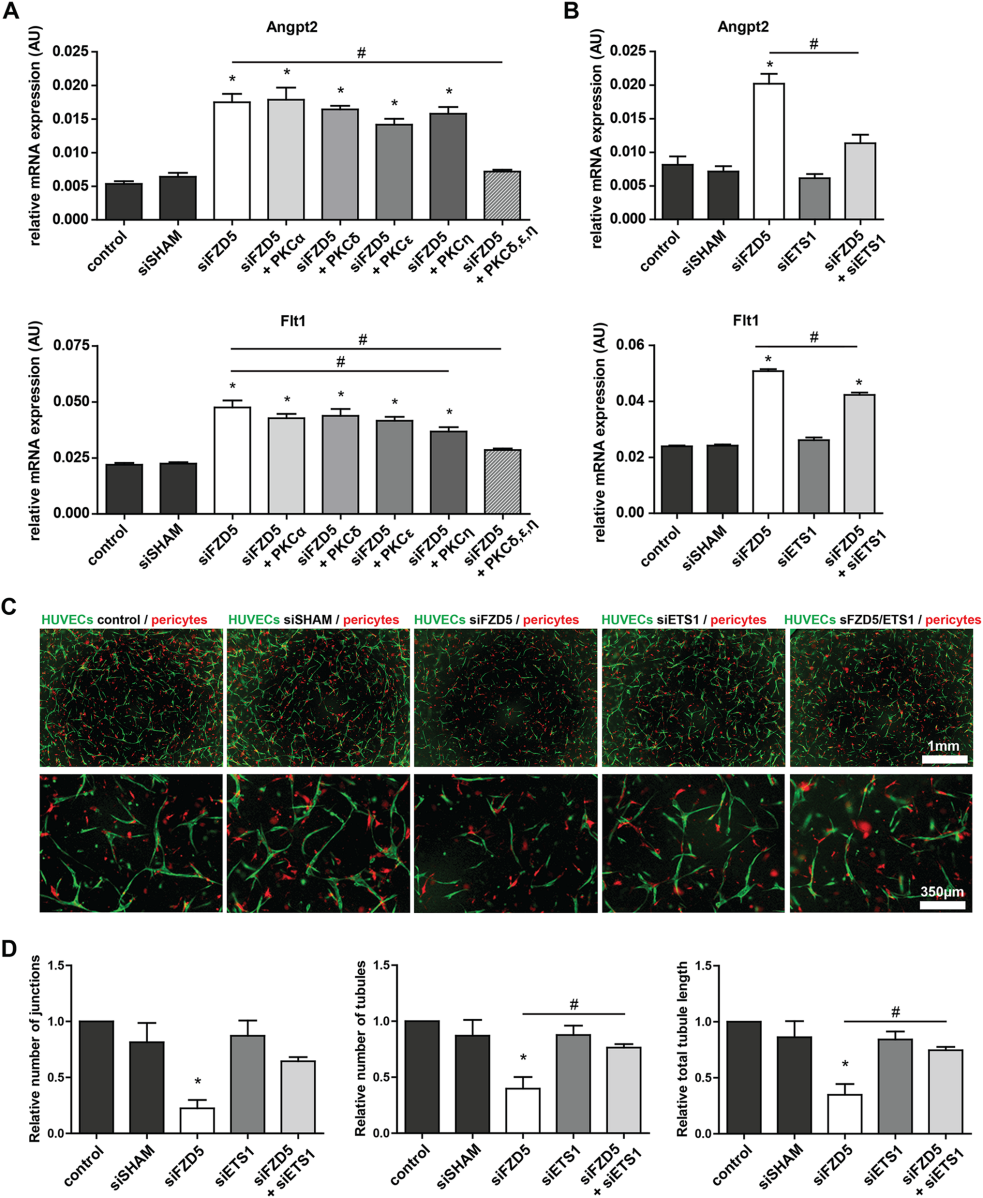
Fzd5 knockdown-induced upregulation of Angpt2 and Flt1 expression is mediated via enhanced PKC and Ets1 signaling. **a** QPCR results showing expression levels of Angpt2 and Flt1 in HUVECs after knockdown of Fzd5 alone, in combination with knockdown of different PKC isoforms, and in combination with knockdown of all novel PKC isoforms (PKCδ,ε,η), 48 h post-transfection. *N* = 4, *P < 0.05 compared to control and siSHAM condition, ^#^*P* < 0.05 as indicated in graph. **b** QPCR results of Angpt2 and Flt1 expression in HUVECs, 72 h post-transfection, with and without knockdown of transcription factor Ets1, a downstream target of PKC. *N* = 4, *P < 0.05 compared to control and siSHAM condition, ^#^*P* < 0.05 as indicated in graph. **c** Representative fluorescent microscope images of GFP-labeled HUVECs (green) in co-culture with dsRED-labeled pericytes (red) in a 3D collagen matrix during vascular formation. Shown are the results at day 5 of non-transfected control, siSHAM, siFzd5, siEts1 and the combined knockdown of Fzd5 and Ets1. Scale bar in the upper row represents 1 mm, in the bottom row 350 µm. **d** Bar graphs show the quantified results of the co-culture assay. Shown are the total tubule length, and the number of endothelial junctions and tubules after 5 days. *N* = 6, *P < 0.05 compared to control and siSHAM condition, ^#^*P* < 0.05 as indicated in graph

Previously, it was demonstrated that Wnt signal transduction also involves PKC [25–27]. PKCs are part of a kinase family with a diverse range of potential downstream targets. To verify whether Fzd5 knockdown-induced upregulation of Flt1 and Angpt2 depended on activation of PKC, HUVECs were treated with the PKC inhibitor Staurosporine in the concentration range of 5–20 nM, as not all different PKC family members are equally inhibited at similar concentrations. Interestingly, both Angpt2 and Flt1 overexpression induced by Fzd5 knockdown were dose-dependently reduced by PKC inhibition compared to control and siSHAM condition (Supplemental Fig. 5A). Since HUVECs express multiple PKC isoforms [28, 29], PKC expression was knocked down by siRNA to interrogate which isoform mediated the observed upregulation of Angpt2 and Flt1. Individual PKC isoform knockdown only had a minor effect on the Fzd5 knockdown-induced overexpression of the anti-angiogenic factors, whereas combined knockdown of the novel PKCs (nPKCs) completely attenuated the upregulation of Angpt2 and Flt1 (Fig. 7a, Supplemental Fig. 5B).

PKC signaling can induce elevated synthesis of the transcription factor Protein C-ets1 (Ets1) [30, 31], which has binding sites in the promoter regions of both Angpt2 and Flt1 [32, 33]. Ets1 was significantly upregulated in the absence of Fzd5, which was orchestrated by PKC (Supplemental Fig. 5C). Involvement of Ets1 in transcriptional regulation of Angpt2 and Flt1 was evaluated in the Fzd5 knockdown condition using a double knockdown of both Fzd5 and Ets1. Knockdown of Ets1 alone had no effect on the expression of Flt1 and Angpt2 compared to control and siSHAM condition, indicating no active transcription regulation of these two genes by Ets1 in control conditions. However, knockdown of Ets1 in Fzd5-silenced HUVECs fully inhibited upregulation of Angpt2 and partially inhibited the upregulation of Flt1 when compared to Fzd5-silenced controls (Fig. 7b). The involvement of Ets1-induced transcription was further substantiated by a similar Ets1-dependent upregulation of Matrix metalloproteinase 1 (MMP1), a verified endothelial target gene of Ets1 (Supplemental Fig. 6A, B) [34]. To evaluate if the anti-angiogenic phenotype of Fzd5 silencing observed in the 3D angiogenesis co-culture assay was mediated via this pathway, Ets1 was silenced in GFP-labeled HUVECs. Analysis of the 3D co-culture results demonstrated that inhibition of Ets1 in the Fzd5 knockdown condition partly rescued the Fzd5 knockdown-mediated reduction of endothelial tubule formation (Fig. 7c, d).

## Discussion

The main findings of the current study are (1) endothelial Fzd5 expression is essential for vascular formation, as shown in a 3D co-culture assay. (2) Fzd5 silencing inhibits EC proliferation and migration. (3) Endothelial loss of Fzd5 expression does not interfere with endogenous canonical Wnt signaling. (4) Fzd5 knockdown leads to increased expression of vascular regression-associated factors Flt1 and Angpt2, independent of both the non-canonical Wnt/Ca^2+^-mediated activation of NFAT and PCP-mediated activation JNK. (5) Inhibition of nPKC signaling, as well as knockdown of the PKC target Ets1 suppressed the upregulation of Flt1 and Angpt2 in the absence of Fzd5. The Ets1 knockdown intervention also partially rescued the Fzd5 knockdown-induced inhibitory effect on new vessel formation.

Previously, it was reported that Fzd5 is indispensable for murine embryogenesis [5]. Fzd5 knockout embryos died in utero from severe defects in yolk sac and placenta vascularization. Using trophoblast-specific Fzd5 knockout mice, Lu et al. reported that the observed phenotype in the Fzd5 full knockout placenta was partly initiated by a defect in chorionic branching morphogenesis [35]. As defective branching morphogenesis of the chorion of these mice resulted in a smaller placental labyrinth layer compared to wild-type littermates, it remained difficult to distinguish whether the placental defects observed in the Fzd5 full knockout mice were indeed vascular related, or the outcome of proportional growth limitations resulting from the reduced villous volume. In our study, we demonstrated that endothelial knockdown of Fzd5 in vitro leads to a severe reduction in vascular tubule formation in a 3D co-culture model, thereby providing evidence for the direct role of Fzd5 in new vessel growth.

The most detailed described Fzd/Wnt signaling cascade is the canonical or β-catenin-dependent pathway. Without stimulation of the canonical pathway, β-catenin is degraded by a destruction complex consisting of Axin, Glycogen Synthase Kinase 3ß, Adenomatous Polyposis Coli, and Casein Kinase 1α. Upon binding of Wnt ligands to a Fzd receptor in the presence of the co-receptor Lrp5 or Lrp6, a conformation change in Lrp extracts Axin away from the destruction complex, leading to an increase in intracellular β-catenin levels. When translocated into the nucleus, β-catenin binds to the TCF/Lef complex and promotes the expression of β-catenin target genes [6–8]. Knockdown of a Fzd receptor could both have an inhibiting effect on this pathway, due to a reduction in receptors capable of transducing a signal for downstream signaling cascade activation, and an activating effect, either due to impaired inhibitory crosstalk between the individual pathways or via alternative receptor binding by the Wnt ligand [18, 19]. Involvement of Fzd5 in this canonical pathway appears to be tissue dependent. Steinhart et al. recently demonstrated that canonical Wnt signaling via Fzd5 was involved in pancreatic tumor growth and Caricasole et al. reported enhanced β-catenin-mediated signaling upon Wnt7a interaction with both Fzd5 and Lrp6 in the rat pheochromocytoma cell line PC12 [36, 37]. In the mouse optic vesicle, however, no evidence suggests that Fzd5 activates or suppresses canonical Wnt signaling [38, 39]. Our analysis of endogenous canonical Fzd/Wnt signaling suggests that Fzd5 is not involved in Wnt β-catenin signaling in ECs.

In contrast to the β-catenin target genes, expression levels of Angpt2 and Flt1 were significantly upregulated in HUVECs with suppressed Fzd5 expression. Angpt2 by itself is known to have a positive effect on neovessel formation, as it is involved in pericyte detachment and destabilization of the endothelium to potentiate the actions of pro-angiogenic factors [40, 41]. However, in the absence of VEGFa, or in the presence of an increased expression of Flt1, a decoy receptor for VEGFa, Angpt2 is known to induce vascular regression [9–11]. Both Angpt2 and Flt1 are potential downstream target genes of the non-canonical Fzd/Wnt signaling pathways. Upon stimulation of the Fzd/Wnt/Ca^2+^ pathway, activation of phospholipase C leads to cleavage of the membrane component PIP2 into DAG and IP3. When IP3 binds to its receptor on the endoplasmic reticulum, Ca^2+^ is released in the cytosol, activating the transcription factor NFAT via Calcineurin [6]. In recent studies, Flt1 and Angpt2 were shown to be transcriptional targets of NFAT [42, 43]. Like Angpt2 and Flt1, the endogenous NFAT inhibitor DSCR1 is also a verified target of the transcription factor NFAT [20, 21], yet our data showed that the expression level of DSCR1 remained stable after knockdown of Fzd5. More important, our experiments demonstrated that inhibition of NFAT activation with CsA after endothelial knockdown of Fzd5 did not inhibit the upregulation of Angpt2 and Flt1, suggesting that the enhanced transcription of these anti-angiogenic factors was not mediated by enhanced activity of NFAT. Alternatively, stimulation of the Fzd/Wnt/PCP pathway can also induce the transcription of Flt1 and Angpt2 via GTPase-mediated activation of JNK, which eventually activates c-JUN-based transcription [6]. Multiple studies provided evidence for transcriptional regulation of Flt1 and Angpt2 either by c-JUN alone, or by the transcription complex AP-1 involving c-JUN [22, 23]. Our data indicated that Fzd5 knockdown led to an increase in JNK phosphorylation, but no increase in c-JUN phosphorylation was observed. In addition, inhibition of JNK activity with SP600125 ruled out the involvement of the PCP-JNK signal transduction axis as causal factor for the enhanced expression of vascular regression-associated factors Angpt2 and Flt1 in ECs with Fzd5 knockdown, as upregulation of these factors remained evident. In future studies, however, it remains of interest to further dissect the relevance of this altered JNK signaling in the absence of Fzd5.

Multiple reports have previously suggested a role for PKC involvement in Fzd/Wnt signaling [25–27]. Staurosporine, as well as siRNA-mediated knockdown of nPKCs inhibited the upregulation of Angpt2 and Flt1 in HUVECs with suppressed expression of Fzd5, indicating the involvement of PKC signaling in the transcriptional regulation of these genes in Fzd5-silenced ECs. The promoter regions of both Angpt2 and Flt1 contain binding sites of the transcription factor Ets1 [32, 33], which was shown by our data to be PKC dependently upregulated in the absence of Fzd5. Our results demonstrate the involvement of enhanced Ets1-mediated transcription of these two genes in Fzd5-silenced ECs, as Ets1 knockdown resulted in a marked repression of Angpt2 and Flt1 expression levels. Another validated endothelial target of PKC/Ets1-mediated transcription, MMP1, which like Angpt2 and Flt1 was previously shown to be involved in vascular regression [34], was also upregulated via Ets1 in the absence of Fzd5. The involvement of Ets1 was further validated using the 3D co-culture model, in which Ets1 knockdown in Fzd5-silenced ECs partially rescued the inhibitory effect on new vessel formation that was observed in Fzd5-silenced conditions. These results indicate a repressing function on PKC/Ets1 signaling by Fzd5 in ECs, leading to reduced expression of vascular regression-associated factors Angpt2 and Flt1.

**Fig. 8:**
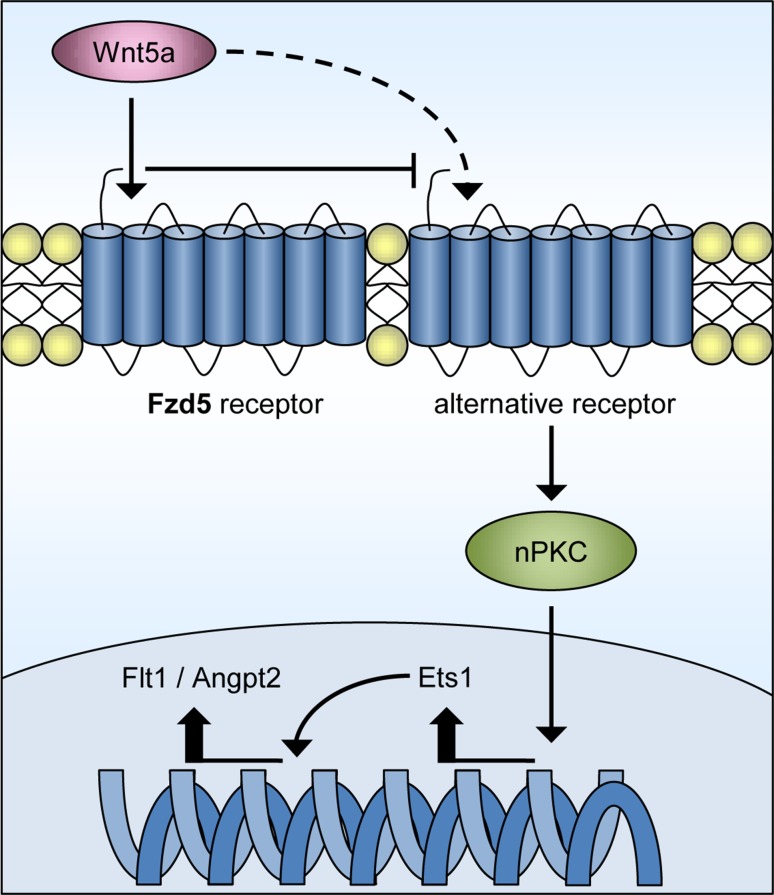
Schematic representation of the proposed model of signaling via Fzd5 in ECs. Our data provide evidence for a new proposed model of signaling in ECs in the absence of Fzd5. Knockdown of this receptor provokes its ligand Wnt5a to signal via an alternative receptor, thereby triggering the activation of nPKC/Ets1-mediated transcription of vascular regression-associated factors, among which Flt1 and Angpt2

In this study, the effect of Fzd5 knockdown on the different Fzd/Wnt signaling routes was studied without the addition of exogenous Wnt factors. HUVECs secrete Wnt factors themselves, among which the typical canonical factor Wnt3 and non-canonical factor Wnt5a. Knockdown of endothelial Fzd5 led to functional defects, as well as differential expression of important genes in the angiogenic process, indicating that lack of Fzd5 interferes with endogenous Fzd/Wnt signaling. The nature of this endogenous signaling in the absence of Fzd5 was shaped by the finding that combined knockdown of Fzd5 and endogenous Wnt5a significantly suppressed Angpt2 and Flt1 upregulation (Supplemental Fig. 7). It was previously demonstrated that Wnt factors induce signaling to a variety of Fzd and non-Fzd receptors, and that binding selectivity is receptor context dependent [13, 44]. As suppression of endogenous Wnt5a signaling partially rescued the Fzd5 knockdown-induced upregulation of Angpt2 and Flt1, our data suggest that endothelial knockdown of Fzd5 provokes its ligand Wnt5a to signal via an alternative receptor, thereby triggering the activation of the observed PKC/Ets1-mediated transcription (Fig. 8). Although our experiments demonstrate that this alternative signaling route via PKC and Ets1 plays an important role in the poor angiogenic phenotype in the absence of Fzd5, the relative contribution of suppressed Fzd5 signaling itself to this phenomenon is yet to be determined. Future studies should also aim to identify the unknown alternative Wnt5a receptor.

The aim of this study was to explore the involvement of Fzd5 in vascular and perivascular biology, which might eventually serve as a foundation for future therapeutic strategies, e.g., in modulating tumor vasculature. A recent genome-wide CRISPR-Cas9 study demonstrated that Fzd5 is a potential druggable target in specific subtypes of pancreatic tumors [36]. Signaling via Fzd5 in these tumor cells was shown to be crucial in β-catenin-mediated proliferation and treatment of these pancreatic adenocarcinoma cells with Fzd5 antibodies led to inhibited cell growth, both in vitro and in xenograft models in vivo. Although these pancreatic adenocarcinoma tumors are not excessively vascularized, they were previously shown to depend on angiogenesis for growth [45, 46]. Our data demonstrate the importance of Fzd5 in ECs during angiogenesis and might imply that targeting the Fzd5 in these types of tumors not only affects the pancreatic adenocarcinoma cells, but could in addition potentially result in beneficial suppression of tumor vascularization.

In conclusion, the current study provides evidence for an important role of endothelial Fzd5 in angiogenesis, thereby providing novel insights in the molecular mechanism causal to the poor angiogenic phenotype in the absence of this receptor.

## Electronic supplementary material

Below is the link to the electronic supplementary material.

Supplementary material 1 (DOCX 2907 KB)
