# Supplementary material for: Endothelial loss of Fzd5 stimulates PKC/Ets1-mediated transcription of Angpt2 and Flt1
Source: Angiogenesis. 2018 May 29;21(4):805–21. doi: 10.1007/s10456-018-9625-6 (PMC6208898; doi:10.1007/s10456-018-9625-6)
Supplement: Supplementary file 1 — Supplementary material 1 (DOCX 2907 KB) [file 10456_2018_9625_MOESM1_ESM.docx]

**Journal:** Angiogenesis

**Endothelial loss of Fzd5 stimulates PKC/Ets1-mediated transcription of Angpt2 and Flt1.**

Maarten M. Brandt^1^, Christian G.M. van Dijk^2^, Ihsane Chrifi^1^, Heleen M. Kool^3^, Petra Burgisser^3^, Laura Louzao-Martinez^2,4^, Jiayi Pei^2^, Robbert J. Rottier^3^, Marianne C. Verhaar^2^, Dirk J. Duncker^1^, Caroline Cheng^1,2^.

*^1^Experimental Cardiology, Department of Cardiology, Thoraxcenter Erasmus University Medical Center, Rotterdam, The Netherlands; ^2^Department of Nephrology and Hypertension, Division of Internal Medicine and Dermatology, University Medical Center Utrecht, Utrecht, The Netherlands; ^3^Sophia Children's Hospital Department of Pediatric Surgery of the Erasmus Medical Center, Rotterdam, The Netherlands.*

**Corresponding author:**

Caroline Cheng, PhD

University Medical Center Utrecht

PO Box 85500, 3508 GA Utrecht, The Netherlands

T: +31 (0)-88-7557329

E-mail: K.L.Cheng-2@umcutrecht.nl

**Supplemental figures**


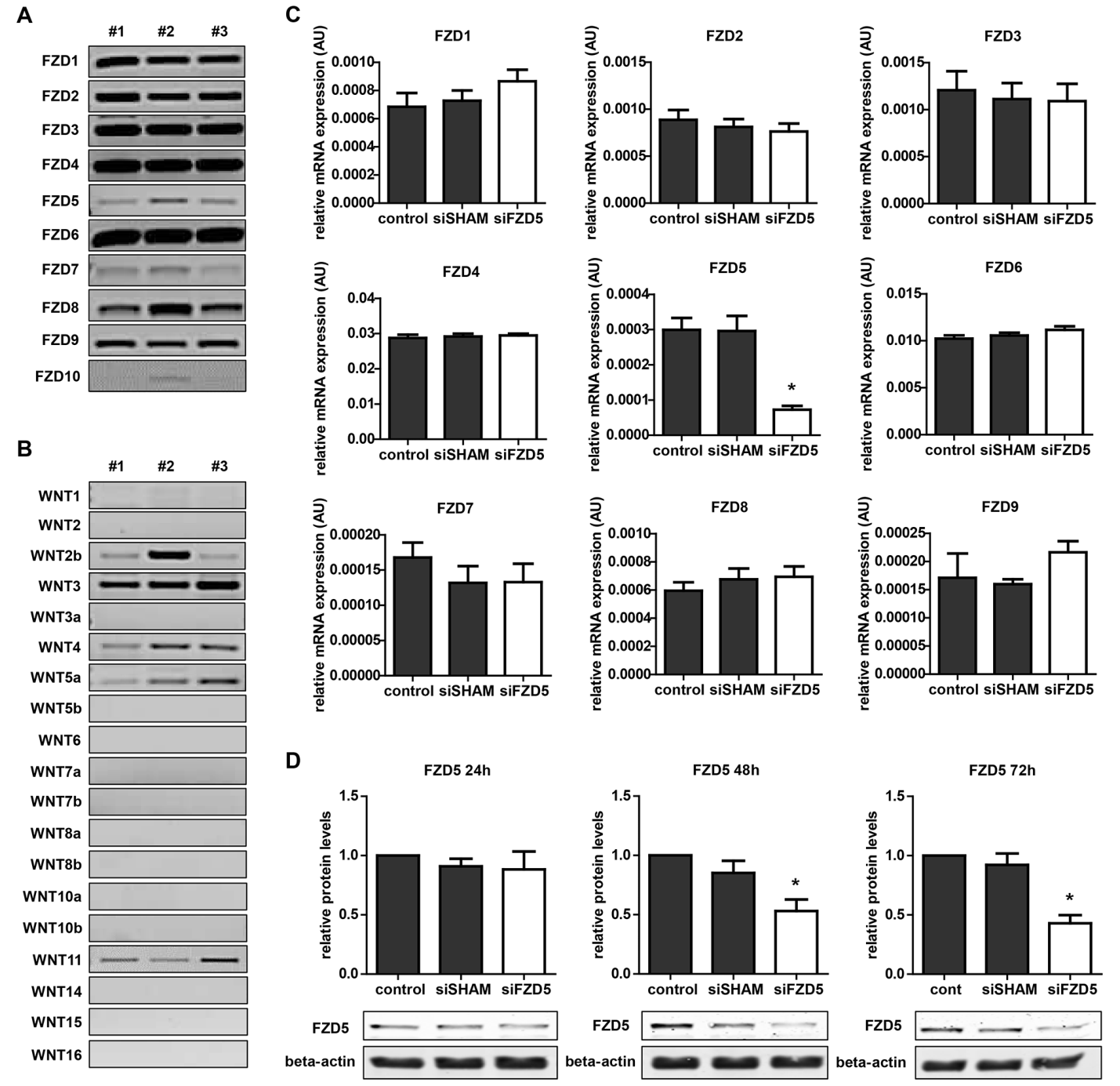


**Supplemental figure 1: Fzd5 siRNA induced a significant and specific knockdown of Fzd5. (A)** PCR results showing expression levels of Fzd receptors in HUVECs. N=3 (#1-3 indicate these replicates). **(B)** PCR results showing expression levels of Wnt ligands in HUVECs. N=3 (#1-3 indicate these replicates). **(C)** QPCR results of expression levels of all Fzd receptors expressed in HUVECs (Fzd1-9) in the different conditions 20 hours post transfection. N=7, *P<0.05 compared to control and siSHAM condition. **(D)** Representative Western blot of Fzd5 and β-actin levels 72 hours post transfection. N=5.


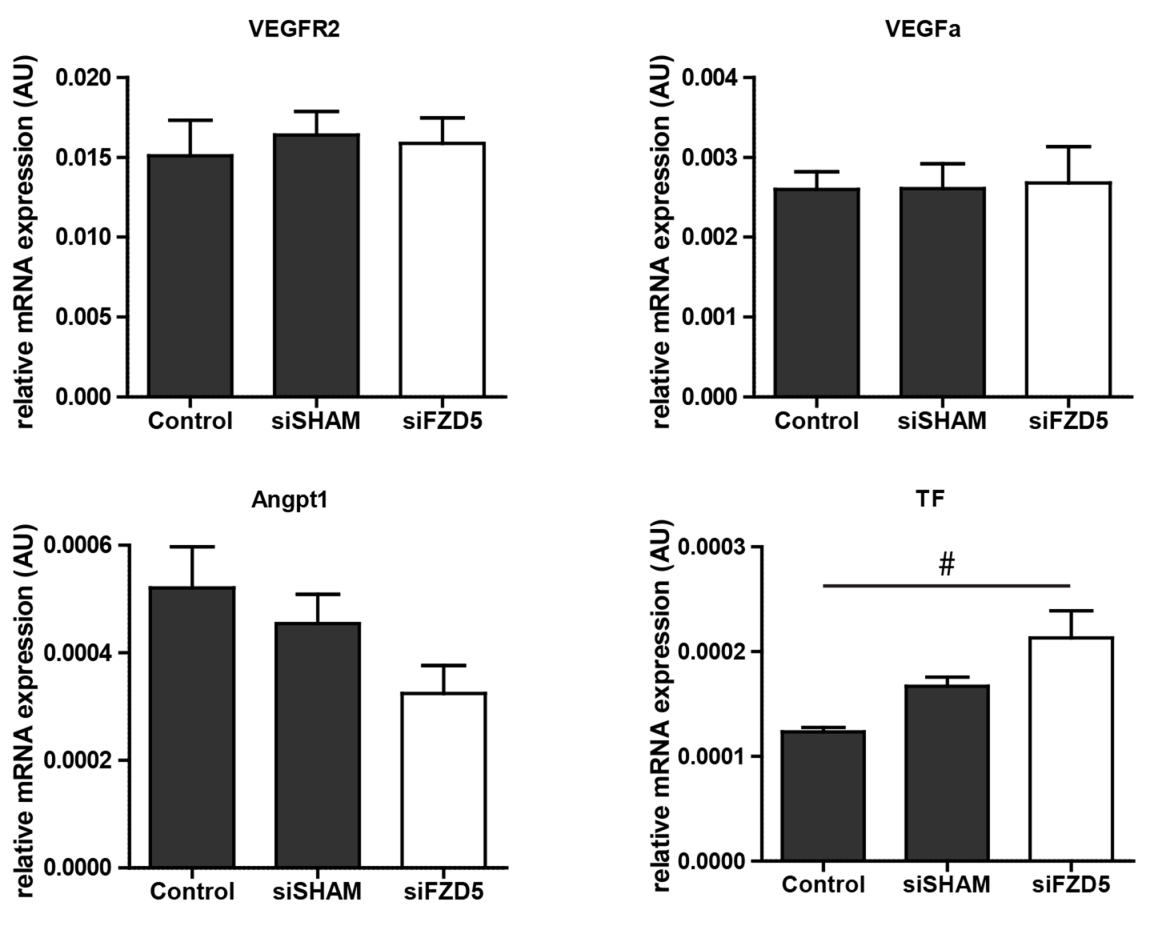


**Supplemental figure 2: Effect of Fzd5 knockdown on important angiogenic regulators.** qPCR results showing expression levels of VEGFr2, VEGFa, Angpt1 and TF in the different conditions 72 hours post transfection. N=5, #P<0.05 compared to untreated control condition.
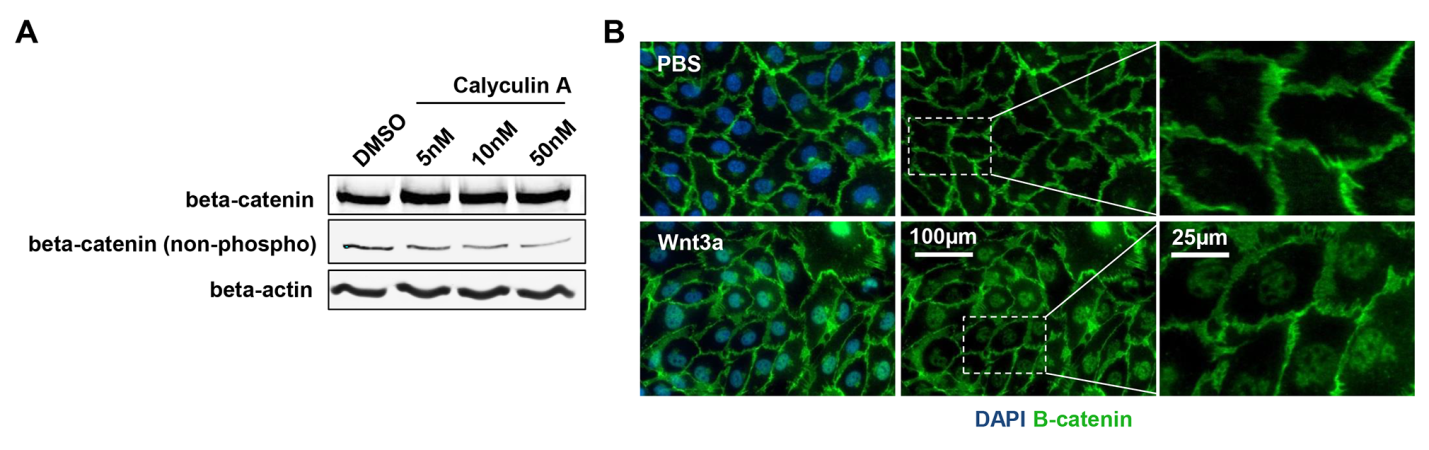
 **Supplemental figure 3: Positive control experiments for β-catenin Western blot and immunofluorescent staining. (A)** Representative Western blot of total β-catenin, non-phospho β-catenin, and β-actin levels in HUVECs after 30 min stimulation with DMSO or different concentrations of the phosphatase inhibitor Calyculin A (positive control for verification of phosphorylation status). **(B)** Representative immunofluorescent staining of β-catenin (green) in HUVECs after stimulation with PBS or recombinant Wnt3a. Scale bars represent 100µm and 25µm.

**
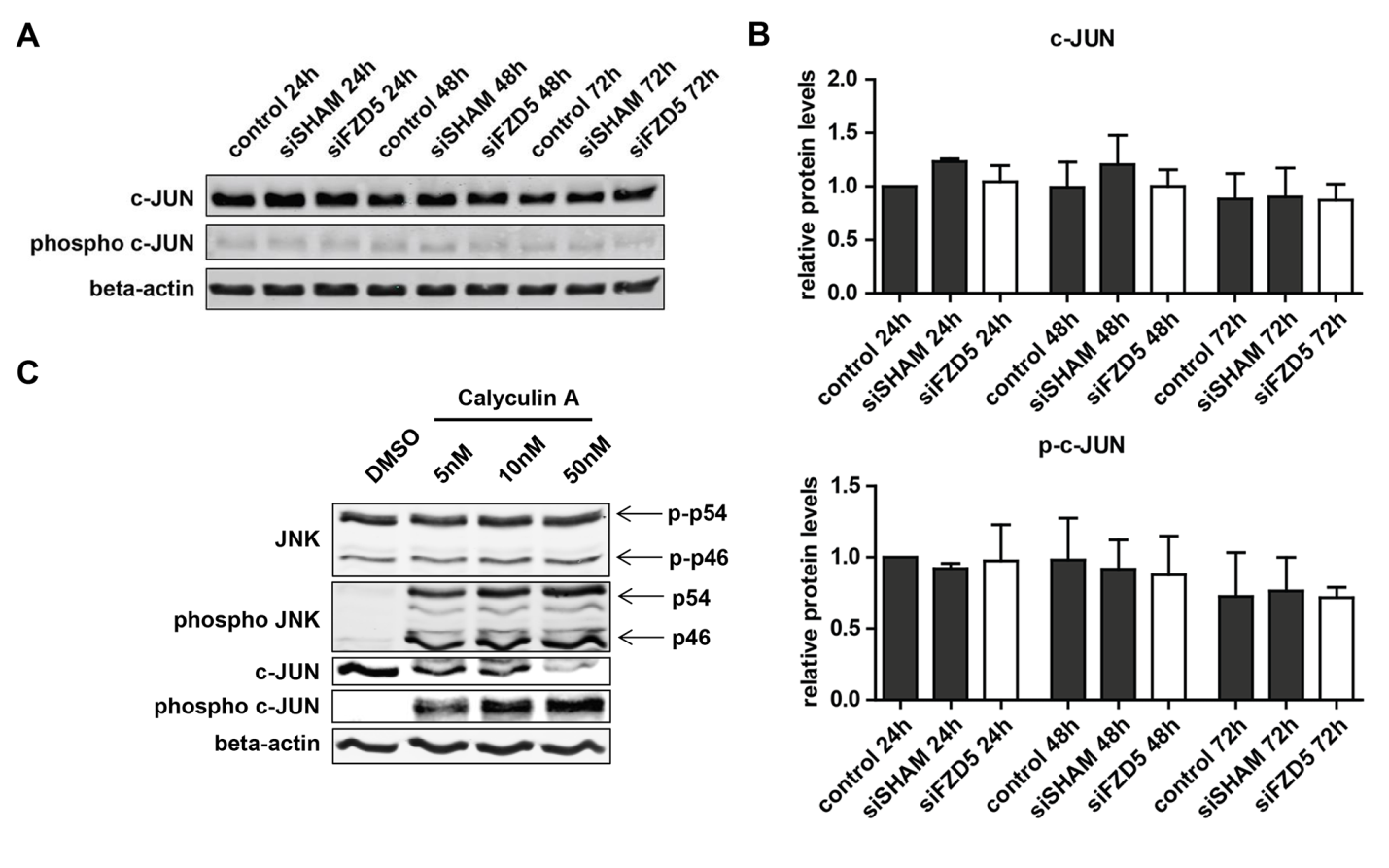
**

**Supplemental figure 4: Fzd5 knockdown did not affect c-JUN phosphorylation. (A)** Representative Western blot of total c-JUN, phospho c-JUN and β-actin levels at different time points post-transfection. **(B)** Quantified results of c-JUN and phospho c-JUN Western blot. Shown are (phospho) c-JUN levels relative to β-actin loading control. N=3, no significance. **(C)** Representative Western blot of total JNK, phospho JNK, total c-JUN, phospho c-JUN and β-actin levels in HUVECs after 30 min stimulation with DMSO or different concentrations of the phosphatase inhibitor Calyculin A (positive control for verification of phosphorylation status).

**
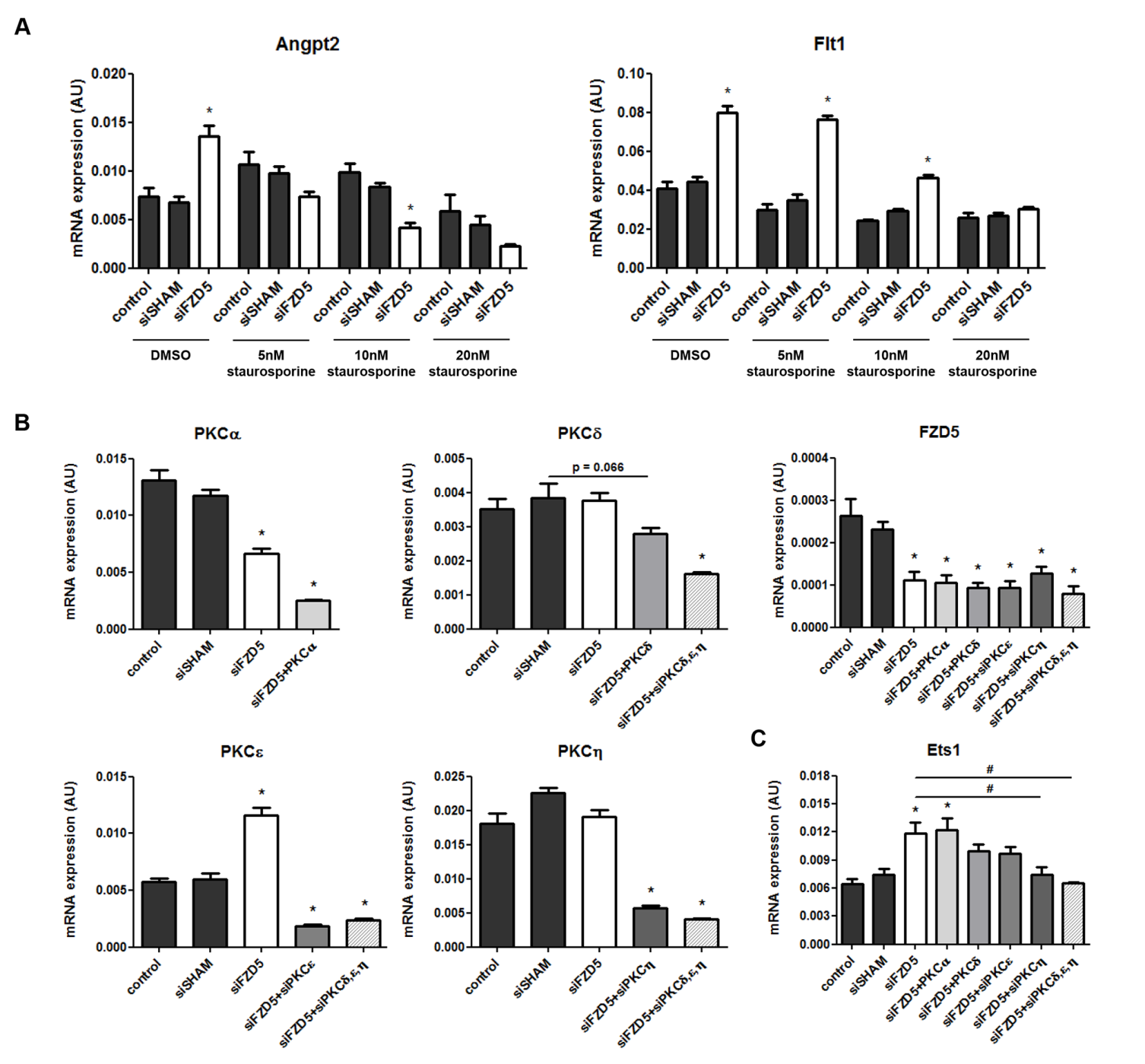
**

**Supplemental figure 5: Upregulation of Angpt2 and Flt1 in HUVECs with suppressed Fzd5 expression via novel PKC signaling. (A)** QPCR results of Angpt2 and Flt1 in response to treatment to PKC inhibitor staurosporine (0-20nM), supplemented 48 hours post transfection to the different conditions. N=4, *P<0.05 compared to control and siSHAM condition within comparable conditions (two-way ANOVA followed by Bonferroni post hoc test). **(B)** QPCR results showing expression levels of conventional PKC isoform PKCα, novel PKC isoforms PKCδ, PKCε, and PKCη, Fzd5, and transcription factor Ets1 **(C)** in HUVECs after knockdown of Fzd5 alone, in combination with different PKC isoforms, and in combination with all novel PKC isoforms (PKCδ,ε,η), 48 hours post transfection. N=4, *P<0.05 compared to control and siSHAM condition, ^#^P<0.05 as indicated in graph.


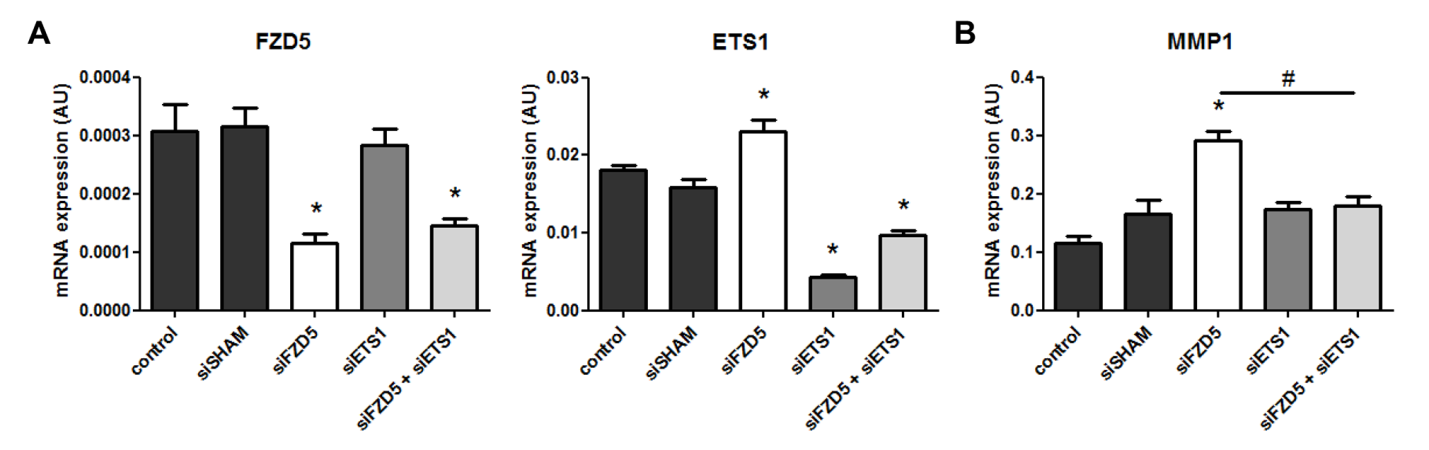
**Supplemental figure 6:** **Fzd5 and Ets1 knockdown validation and Ets1 dependent MMP1 expression in absence of Fzd5.** qPCR results showing expression levels of Fzd5 and Ets1 **(A)**, and Ets1 target gene MMP1 **(B)** in HUVECs after knockdown of Fzd5, Ets1, and in a combined knockdown of Fzd5 and Ets1, 72 hours post transfection. N=4, *P<0.05 compared to control and siSHAM condition, ^#^P<0.05 as indicated in graph.

**
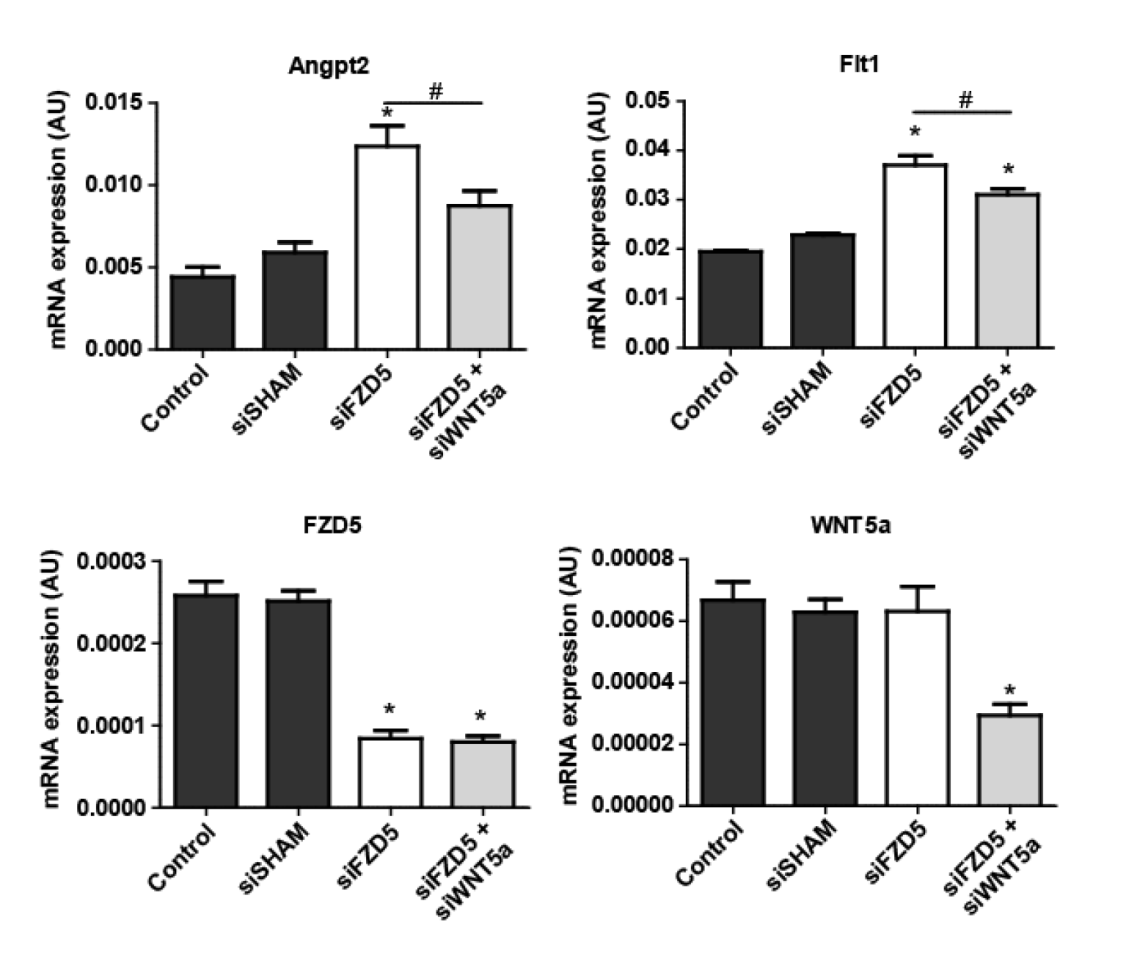
**

**Supplemental figure 7:** **Endogenous endothelial Wnt5a expression triggers Angpt2 and Flt1 upregulation in absence of Fzd5.** qPCR results showing expression levels of Angpt2, Flt1, Fzd5 and Wnt5a in HUVECs after knockdown of Fzd5 alone or in combination with Wnt5a, 72 hours post transfection. N=11, *P<0.05 compared to control and siSHAM condition, ^#^P<0.05 as indicated in graph.
